# A photo-switchable assay system for dendrite degeneration and repair in *Drosophila melanogaster*

**DOI:** 10.1073/pnas.2204577119

**Published:** 2022-08-15

**Authors:** Han-Hsuan Liu, Chien-Hsiang Hsu, Lily Y. Jan, Yuh-Nung Jan

**Affiliations:** ^a^Department of Physiology, University of California, San Francisco, CA 94143;; ^b^Department of Biochemistry and Biophysics, University of California, San Francisco, CA 94143;; ^c^HHMI, University of California, San Francisco, CA 94143;; ^d^Department of Pharmaceutical Chemistry, University of California, San Francisco, CA 94143

**Keywords:** dendrite degeneration, dendrite repair, dendrite regeneration, *Drosophila*, Wallerian degeneration slow protein

## Abstract

Neurodegeneration arising from aging, injury, or diseases has devastating health consequences. Whereas neuronal survival and axon degeneration have been studied extensively, much less is known about how neurodegeneration affects dendrites, in part due to the limited assay systems available. To develop an assay for dendrite degeneration and repair, we used photo-switchable caspase-3 (caspase-Light–Oxygen–Voltage-sensing [caspase-LOV]) in peripheral class 4 dendrite arborization (c4da) neurons to induce graded neurodegeneration by adjusting illumination duration during development and adulthood in *Drosophila melanogaster*. We found that both developing and mature c4da neurons were able to survive while sustaining mild neurodegeneration induced by moderate caspase-LOV activation. Further, we observed active dendrite addition and dendrite regeneration in developing and mature c4da neurons, respectively. Using this assay, we found that the mouse Wallerian degeneration slow (Wld^S^) protein can protect c4da neurons from caspase-LOV–induced dendrite degeneration and cell death. Furthermore, our data show that Wld^S^ can reduce dendrite elimination without affecting dendrite addition. In summary, we successfully established a photo-switchable assay system in both developing and mature neurons and used Wld^S^ as a test case to study the mechanisms underlying dendrite regeneration and repair.

## Significance

We introduced a versatile system for photo-switchable caspase-3 activation in developing and mature *Drosophila* dendrite arborization neurons to induce degeneration and monitored ensuing dendrite degeneration, protection, repair, regeneration, and cell death. Using this assay system, we observed the protection afforded by Wallerian degeneration slow (Wld^S^) upon photo-switchable caspase-3–induced neurodegeneration and further demonstrated that Wld^S^ does not improve dendrite regrowth or regeneration in development and adulthood, respectively. Because of the ease and flexibility to systematically induce neuronal injury, this assay system should facilitate uncovering the underlying cellular and molecular mechanisms by doing genetic screens. This photo-switchable assay system can provide physiologically relevant insights because caspase-3 is involved in the developmental pruning of axon and dendrite, injury-induced neurodegeneration, and neurodegenerative diseases.

Neurodegeneration places tremendous burdens on both patients and society at large. While much progress has been made in the study of neuronal survival and axon degeneration, how dendrites respond to injuries or neurodegeneration is much less well understood. Aging, neurological disorders, traumatic brain injury, and other insults could result in dendrite degeneration (1–5). The deleterious changes in dendrite structures impair how neurons receive and process information, likely causing major deficits in neurological functions (3, 4). Elucidating the underlying mechanisms of dendrite degeneration and repair will help us to find ways to reduce damage and facilitate recovery with important clinical implications. To better understand whether dendrite degeneration can be attenuated and to what extent dendrites are capable of repair, we need to develop physiologically relevant and reliable in vivo neurodegeneration models.

*Drosophila* dendrite arborization (da) neurons are well suited for studying dendrite development, degeneration, and repair. They are sensory neurons in the body wall, and the confinement of their dendrites in a primarily two-dimensional space is conducive to live imaging (6). Based on the dendrite arbor complexity, da neurons are grouped into four classes, with class 4 dendrite arborization (c4da) neurons displaying the most complex dendrite arbors (7). Studies that use laser ablation to sever dendrites from the c4da, c3da, and c1da neuron somata have shown that dendrites can repair themselves and have provided valuable information suggesting that the repair process depends on kinases, electrical activity, extracellular environment, microRNA, and kinetochore proteins (8–14). However, the harsh injury caused by dendrite severing is likely more severe and abrupt as compared with neurodegeneration induced by neurological disorders, traumatic brain injury, aging, and other insults. Moreover, laser ablation is labor intensive and hence, is not suitable for high-throughput screening designed to uncover novel mechanisms. To gain insights into how dendrites degenerate and repair, it is desirable to develop an alternative neurodegeneration model that can better simulate how a neuron responds to the insults that it may encounter in its lifetime.

Many conditions can induce neurodegeneration. In this study, we used caspase-3, which acts downstream of various insults, as a switch to initiate neurodegeneration. Activation of caspase-3, an executor for apoptotic cell death, has been observed in neurons exposed to insults, such as injury, neurotoxins, and neurodegenerative diseases (15, 16). There are also circumstances where following caspase-3 activation, neurons stay alive and display degeneration or partial remodeling in dendrites or axons (17–21). These observations suggest that caspase-3 could be used as a way to introduce damage to dendrites systematically to elicit neurodegeneration. A recently developed photo-switchable caspase-3 provides opportunities to test whether a controllable caspase-3 could be a versatile tool to induce neurodegeneration with outcomes ranging from apoptosis to repair (22). In this system, a Light–Oxygen–Voltage-sensing (LOV) domain is inserted into the intersubunit linker of the human caspase-3 (22). Illumination with 450-nm light would expand the LOV domain and activate this photo-switchable caspase-3, caspase-LOV. The activation only lasts for the duration of illumination. This reversible feature of caspase-LOV makes it possible to adjust the degree of caspase-3 activity during a specific time window (22). The duration of illumination is known to correlate with the amount of caspase-3 activity, and hours of illumination can effectively induce dendrite degeneration followed by apoptosis in several types of cells, including c4da neurons (22).

Neuronal expression of the mouse Wallerian degeneration slow (Wld^S^) protein was found to delay Wallerian degeneration, which is an evolutionarily conserved process to clear distal axons after axon injury, in both mice and flies (23–25). Indeed, expression of Wld^S^ can also protect axon degeneration or neuronal survival in several models of neurodegeneration (26, 27). Recently, the roles of Wld^S^ in dendrites have started to be revealed. Expression of Wld^S^ reduces dendrite pruning during metamorphosis and dendrite degeneration induced by laser-severing injury, glaucoma, or phosphatidylserine (PS) exposure (28–32). Whether Wld^S^ can improve dendrite repair or regeneration remains an open question.

To elucidate the cellular mechanism of dendrite degeneration and repair, we adjusted the extent of caspase-LOV activation to induce graded dendrite degeneration in *Drosophila* c4da neurons and monitored their repair process following degeneration. We found that both larval and mature adult c4da neurons can survive and continue adding new dendrite tips following attenuated activation of caspase-LOV. We tested the role of Wld^S^ in caspase-LOV–induced dendrite degeneration and found that expression of Wld^S^ in c4da neurons can retain longer and more numerous dendrites following dendrite degeneration. The greater number of dendrite tips of neurons with the expression of Wld^S^ arose from a reduction of dendrite elimination rather than changes in dendrite additions for both developing and mature neurons. Our assay system provides a versatile platform to study dendrite degeneration, protection, repair, and regeneration during the repair process following insults in the developing as well as mature neurons.

## Results

### Caspase-LOV Activation of Different Durations in Larval Sensory Neurons Initiates Graded Dendrite Degeneration Followed by Repair or Cell Death.

Among larval da neurons, c4da neurons display the most complex dendrite structures (7). Their dendrites actively grow in length, scale in size to extend coverage area, and continue adding new tips throughout the larval development (7, 33, 34). In this study, we sought to determine to what extent c4da neurons can recover from degeneration following transient activation of a photo-switchable caspase-3, caspase-LOV.

**Fig. 1.**
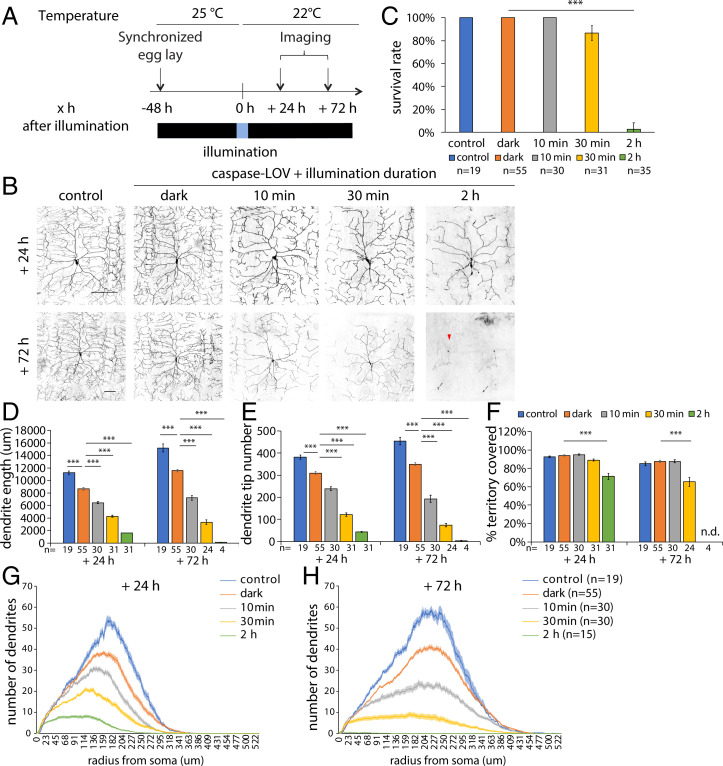
Caspase-LOV activation initiates graded dendrite degeneration in larval c4da neurons. (*A*) Protocol to illuminate and image larval c4da neurons expressing just tdTOM (control) or tdTOM and caspase-LOV using ppk-GAL4. Larvae were kept in the dark all the time (control; dark) or kept in the dark and illuminated at 48 h AEL for different durations. The same neurons were imaged at 24 and 72 h following illumination. (*B*) Representative images of c4da neurons from larva without caspase-LOV and kept in the dark (control), with caspase-LOV and kept in the dark (dark), or with caspase-LOV and illuminated for different durations. Neurons were imaged at 24 h (+24 h; *Upper*) and 72 h (+72 h; *Lower*) after illumination started. The red arrowhead indicates the soma of the same neuron imaged at 24 h. (Scale bars: 100 μm.) (*C*) Survival rates of c4da neurons expressing caspase-LOV decreased when illumination was extended. Survival of neurons was counted 72 h after illumination. The Kruskal–Wallis rank-sum test with Dunn’s post hoc test was further adjusted by the Benjamini–Hochberg False Discovery Rate (FDR) method for multiple independent samples. (*D*–*F*) Quantifications of dendrite structures of survived c4da neurons following caspase-LOV activation, including total dendrite length (*D*), total dendrite tip numbers (*E*), and percentage of territory covered (*F*). The skeletal dendrite structures were predicted by in-house built deep learning models with the quantifications carried out using a python script. (*G* and *H*) Sholl analysis of dendrite complexity 24 h (*G*) and 72 h (*H*) after illumination. All conditions are significantly different from each other (*P* < 0.01). One-way ANOVA with Tukey’s post hoc test was used for multiple comparisons in *D*–*H*. Error bars represent ± SEM (*C*–*F*) or are in the shaded areas (*G* and *H*). *n* = 19 to 55 neurons for each experimental condition and time point as noted. n.d., not detected. ***P < 0.001.

Given that activation of caspase-LOV can be easily controlled by adjusting the intensity and the duration of illumination, we began our study by monitoring dendrite degeneration following caspase-LOV activation for 2 h, 30 min, or 10 min. We used membrane-tethered tdTomato (UAS-CD4-tdTOM) driven by the ppk-GAL4 to label c4da neurons for visualization of individual dendrite arbors. Freely moving larvae were illuminated before being transferred back to a dark environment following the protocol described in Fig. 1*A*. We performed time-lapse imaging to monitor the dendrite structure of the same c4da neuron, ddaC, at 24 and 72 h following illumination (Fig. 1*A*). The 24- and 72-h imaging time points provide snapshots for the early and late stages of caspase-3–induced dendrite degeneration and subsequent repair, as indications for the acute and continuing response to the degeneration, respectively.

**Fig. 2.**
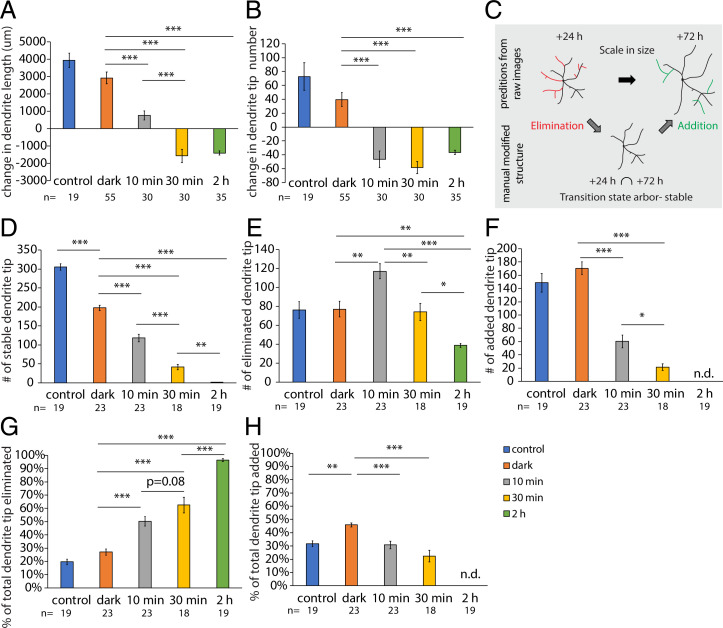
Dendrite addition and elimination occur simultaneously following mild caspase-LOV activation. (*A* and *B*) Quantifications of changes in dendrite length (*A*) and dendrite tip numbers (*B*) of c4da neurons during the 24- to 72-h period after caspase-LOV activation. c4da neurons expressing caspase-LOV decrease growth in dendrite length and dendrite tip numbers as illumination is extended. (*C*) Illustration of the elimination and addition of dendrites that happened over the degeneration and repair process. (*D*–*F*) Quantifications for the number of stable (*D*), eliminated (*E*), and added (*F*) dendrite tips over the 24- to 72-h period following caspase-LOV activation. (*G* and *H*) Quantifications for the percentage of eliminated (*G*) and added (*H*) dendrite tips over the 24- to 72-h period following caspase-LOV activation. The percentage of tips eliminated increased with longer illumination, while the percentage of tips added decreased. One-way ANOVA with Tukey’s post hoc test was used for multiple comparisons in *A*, *B*, and *D*–*H*. Error bars represent ± SEM. *n* = 19 to 23 neurons for each experimental condition and time point as noted. n.d., not detected. *P < 0.05; **P < 0.01; ***P < 0.001.

To facilitate quantification of the complex morphology of c4da neurons in this study, we built a deep learning model based on the U-Net architecture (35), which has been widely used for biomedical image segmentation, including detecting dendrite branch terminals of da neurons (36). We applied our model to automatically segment dendrite structures from microscopy images and retrieve segmentation masks containing the full reconstruction of the dendrite arbors of neurons. Segmentation masks of individual neurons were then used to measure different parameters of neuronal morphology, including total dendrite length, total dendrite tip numbers, percentage of territory covered, and dendrite complexities assessed with Sholl analysis. Dendrite structures segmented by our model were comparable with manual reconstruction and achieved a high Dice coefficient, a commonly used spatial overlap index for evaluating segmentation quality (37) (*SI Appendix*, Fig. S1 *A* and *B*). The overlay images revealed that the majority of the predicted dendrites matched with the manual reconstructions, with the disagreements accounted for by shifts at the *x–y* planes or relatively faint terminal branches (*SI Appendix*, Fig. S1*B*). To further evaluate the model performance, we compared parameters of neuronal morphology measured from model-predicted segmentation with those derived from manual reconstruction by using the images of c4da neurons acquired in Fig. 1, which were not included in the training dataset. With postprocessing to fill in gaps and remove small fragments (*SI Appendix*, Materials and Methods[Sec s16]), we observed high correlations for both tip numbers (*R*^2^ = 0.97) and total dendrite length (*R*^2^ = 0.99) (*SI Appendix*, Fig. S1 *C* and *D*). Caspase-LOV activation lasting 2 h resulted in the survival of only 3% of the neurons at 72 h (Fig. 1 *B* and *C*). Shortening the caspase-LOV activation to 30 min increased the survival rate to 87%, and almost all neurons survived 10-min caspase-LOV activation for at least 72 h (Fig. 1 *B* and *C*). Using the deep learning–based model, we quantified the dendrite structures of c4da neurons and found that activation of caspase-LOV for 2 h resulted in a reduction of total dendrite length, tip numbers, dendrite complexity, and percentage of covered territory 24 and 72 h afterward (Fig. 1 *B* and *D–H*). The total dendrite length, tip numbers, and dendrite complexity decreased progressively with increasing durations of illumination, while the percentage of covered territory was altered at 24 h after 2 h of illumination and 72 h after 30 min of illumination (Fig. 1 *B* and *D–H*). Neurons illuminated for 30 min displayed significantly shorter and fewer dendrites compared with those illuminated for 10 min. The basal activity of caspase-LOV in the dark (dark) led to reduced dendrite arbor length, tip numbers, and dendrite complexity at the 24- and 72-h time points compared with the animals without caspase-LOV expression (control) (Fig. 1 *B*, *D*, *E*, *G*, and *H*). The percentage of territory covered by dendrites was not affected by the expression of caspase-LOV if the animals were kept in the dark (Fig. 1 *B* and *F*). With 30 min and 2 h of caspase-LOV activation, there were overall reduced changes in both dendrite length and tip numbers (Fig. 2 *A* and *B*). Interestingly, there were still increases in the dendrite length 24 to 72 h after the 10-min illumination (Fig. 2*A*), even though the total dendrite tip numbers were reduced (Fig. 2*B*), suggesting that surviving c4da neurons can repair and continue to grow after experiencing mild caspase-LOV activation. Illumination with blue light for 10 min, 30 min, or 2 h did not affect dendrite structures in larval c4da neurons without caspase-LOV at the 24- and 72-h time points (*SI Appendix*, Fig. S2).

### c4da Neurons Add New Branches When Dendrite Degeneration Happens Concurrently.

To monitor the process of dendrite degeneration and dendrite regrowth in the surviving neurons, we performed a dendrite dynamic analysis and quantified the elimination and addition of dendrite branches of c4da neurons over a period of 48 h following caspase-LOV activation. We compared the dendrite structure between the 24- and 72-h time points and used the dendrite arbor at 24 h following illumination as the backbone to generate a “transition state arbor,” which contained only dendrites observed at both 24 and 72 h (Fig. 2*C*). Then, we subtracted the number of tips of the transition state arbor, a measure of the stable dendrites, from that at 24 h to give a measure of the eliminated dendrites (those dendrite branches only observed at 24 h) and from that at 72 h to give a measure of the newly added branches (those dendrite branches only observed at 72 h) (Fig. 2*C*). We found that the number of stable dendrites gradually decreased as the illumination duration was extended (Fig. 2*D*). The numbers of dendrite tips being eliminated or added in c4da neurons expressing caspase-LOV and kept in the dark were comparable with those in c4da neurons not expressing caspase-LOV (Fig. 2 *E* and *F*). The number of eliminated dendrite tips was significantly increased with 10-min illumination, while it decreased with lengthening illumination duration (Fig. 2*E*). Although the number of dendrite tips was significantly reduced following 10 or 30 min of illumination (Fig. 2*B*), there was active dendrite addition in neurons that survived from caspase-LOV activation (Fig. 2*F*). Our data suggest that the reduction in total tip numbers following 10 or 30 min of illumination (Fig. 2*B*) could be attributed to significant increases in elimination (Fig. 2*E*) and decreases in the addition of dendrite branches (Fig. 2*F*).

Removal of the dendritic arbor involves two mechanisms: local degeneration and branch retraction (34, 38, 39). To understand whether dendrites underwent degeneration following the mild caspase-LOV activation, we increased temporal resolution by monitoring the changes in dendrite structure at 24, 32, 48, and 72 h following illumination. We used CD4-tdTom to label c4da dendrites because tdTom-labeled dendrite debris is stable even in phagosomes after being engulfed by the epidermal cells; thus, it can serve as an indication of dendrite breakdown and subsequent engulfment (32). By doing so, we found evidence of dendrite degeneration, including dendrite branch severing, dendrite blebbing, and dendrite branch fragmentation at 24 to 72 h following mild caspase-LOV activation upon 10 and 30 min of illumination (*SI Appendix*, Fig. S3). We also observed dendritic debris near the eliminated dendrites, while the remaining dendrites remained intact (*SI Appendix*, Fig. S3). The dendritic debris did not align with the original dendritic patterns, suggesting that the debris could become engulfed by epidermal cells after dendrite fragmentation (32). Our data strongly support that there was local degeneration following mild caspase-LOV activation.

We also compared how the changes in the numbers of dendrite elimination and addition contribute to the total dendrite tip numbers by calculating the percentage of the eliminated and added dendrite tips by dividing the number of eliminated or added dendrites by the total number of dendrite tips measured at 24 or 72 h, respectively. We found that caspase-LOV expression in c4da neurons without illumination did not change the percentage of eliminated dendrite tips (Fig. 2*G*) but led to a higher percentage of newly added dendrites (Fig. 2*H*) as compared with control c4da neurons without caspase-LOV. With an extended duration of caspase-3 activity, the percentage of eliminated dendrite tips increased, while the percentage of added dendrite tips decreased (Fig. 2 *G* and *H*). Taken together, we found that neurons can survive 10 to 30 min of caspase-LOV activation through illumination, and dendrites of the surviving neurons continued growing in length while dendrite tip addition and elimination took place concurrently.

### Wld^S^ Protects c4da Neurons from Caspase-3–Dependent Dendrite Degeneration.

To facilitate the investigation of genes involved in dendrite degeneration and repair, we generated caspase-tester flies expressing the ppk-tdGFP transgene to monitor the dendrite morphology of c4da neurons with caspase-LOV expressed via ppk-GAL4. These tester flies can be crossed with flies expressing RNA interference (RNAi) or other transgenes of interest. We found that 91% of the neurons survived the 10-min illumination, and the survival rate dropped to 22% following the 30-min illumination (*SI Appendix*, Fig. S4 *A* and *B*). We suspected that the lower survival rate following the 30-min illumination in these experiments reflects the stronger ppk-GAL4 driver with a different insertion site in these caspase-tester flies, as compared with the ppk-GAL4 driver in the experiments shown in Fig. 1. The 10-min caspase-LOV activation reduced the dendrite length and dendrite tip numbers at 24 and 72 h after illumination, and extending caspase-LOV activation to 30 min worsened degeneration (*SI Appendix*, Fig. S4 *A* and *C*–*E*). The percentage of territory covered was not affected in the neurons that survived the 10- or 30-min illumination (*SI Appendix*, Fig. S4 *A* and *E*).

Next, we examined the functions of Wld^S^ in dendrite degeneration and repair. Wld^S^ reduces developmental dendrite pruning as well as dendrite degeneration induced by laser-severing injury, PS exposure, or glaucoma (28–30, 32). Given that Wld^S^ is involved in several types of dendrite degeneration, we wondered if Wld^S^ would be protective in the graded dendrite degeneration induced by caspase-LOV. We were also curious about the role of Wld^S^ in dendrite repair, which has not been addressed in previous reports. To test whether the expression of Wld^S^ plays any role in dendrite repair, we took advantage of the 10-min illumination condition allowing active dendrite repairs following caspase-3–induced degeneration. By including the 30-min illumination condition, we tested the effectiveness of the protection mediated by Wld^S^ upon severe degeneration often leading to cell death.

Using the caspase-tester flies, we examined how Wld^S^ expression might affect caspase-3–dependent dendrite degeneration and repair. To maintain comparable expression levels of UAS–caspase-LOV driven by ppk-GAL4 in neurons with or without Wld^S^ expression, we included the UAS-monomeric infrared fluorescent proteins-2A-Heme Oxygenase 1 Proteins (UAS-mIFP-2A-HO1) transgene instead of UAS-Wld^S^ in the control group. We chose the UAS-mIFP-2A-HO1 transgene as a control because the expression of mIFP-2A-HO1 does not interfere with the imaging of either GFP or tdTOM but can still be visualized under the microscope if needed. Expression of Wld^S^ increased the total dendrite length and tip numbers without altering the percentage of territory covered during early dendrite development (*SI Appendix*, Fig. S5). Wld^S^-expressing neurons retained significantly longer dendrites and more numerous dendrite tips at 72 h following 10 min of caspase-LOV activation, although they were comparable with control neurons at 24 h following illumination (*SI Appendix*, Fig. S6 *A*–*C*). The protection in the dendrite structure afforded by Wld^S^ was already evident at 24 h following 30 min of caspase-LOV activation, as revealed by the longer dendrites and more numerous dendrite tips (*SI Appendix*, Fig. S6 *E*, *G*, and *H*). Wld^S^ expression did not alter the percentage of territory covered following 10- or 30-min caspase-LOV activation (*SI Appendix*, Fig. S6 *D* and *I*). With 30 min of illumination, neuronal survival was enhanced by Wld^S^ expression in c4da neurons (*SI Appendix*, Fig. S6*F*). These results suggest that the expression of Wld^S^ can protect c4da neurons from caspase-3–induced dendrite degeneration and cell death.

### Wld^S^ Reduces Dendrite Elimination in Larval c4da Neurons following Caspase-LOV Activation.

**Fig. 3.**
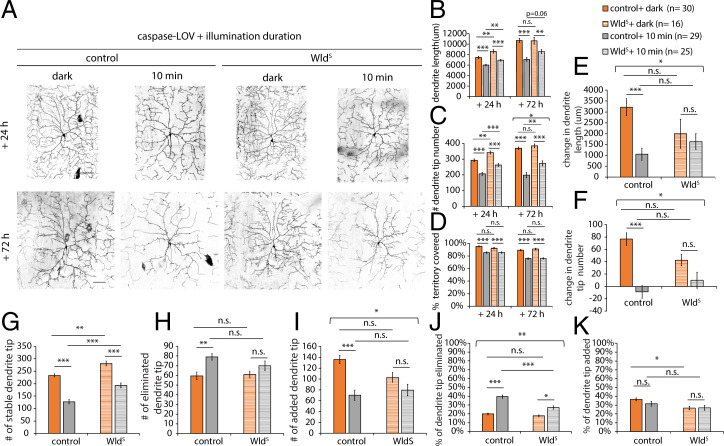
Overexpression of Wld^S^ increased the number of stable dendrite tips and reduced the percentage of dendrite being eliminated following caspase-3–induced dendrite degeneration. (*A*) Representative images of c4da neurons from larvae harboring ppk-GAL4, UAS-tdTOM, UAS–caspase-LOV, and UAS-mIFP-2A-HO1 (control) or UAS-Wld^S^ (Wld^S^). Larvae were kept in the dark or illuminated for 10 min and imaged following the protocol in Fig. 1*A*. The same neurons were imaged twice at 24 and 72 h following illumination. (Scale bars: 100 μm.) (*B*–*D*) Quantifications of dendrite structures of surviving c4da neurons with or without caspase-LOV activation, including total dendrite length (*B*), total dendrite tip numbers (*C*), and percentage of territory covered (*D*). (*E* and *F*) Quantifications of changes in dendrite length (*E*) and dendrite tip numbers (*F*) of c4da neurons during the 24- to 72-h period after caspase-LOV activation. (*G*–*I*) Quantifications for the number of stable (*G*), eliminated (*H*), and added (*I*) dendrite tips over the 24- to 72-h period following caspase-LOV activation. (*J* and *K*) Quantifications for the percentage of eliminated (*J*) and added (*K*) dendrite tips over the 24- to 72-h period following caspase-LOV activation. The percentage of tips eliminated decreased with Wld^S^ expression. Two-way ANOVA for testing the interaction between caspase-LOV activation and Wld^S^ expression was used. Square brackets mark the group that showed a statistically significant interaction. Error bars represent ± SEM. *n* = 16 to 30 neurons as noted. n.s., not significant. *P < 0.05; **P < 0.01; ***P < 0.001.

Wld^S^ can retain more dendrite tips and longer dendrites in the surviving neurons, but it is unclear whether this resulted from reducing dendrite degeneration or improving dendrite repair. To distinguish the contribution of dendrite elimination and addition following caspase-3–induced degeneration, we performed the dendrite dynamic analysis on neurons expressing Wld^S^ that were either kept in the dark or illuminated for 10 min. To track the neuron over time with minimum blue light illumination for imaging GFP, we generated another type of tester flies with ppk-GAL4 driving tdTomato and caspase-LOV and crossed these tester flies with flies harboring UAS-mIFP-2A-HO1 (control) or UAS-Wld^S^. With the ppk-GAL4 driving three instead of two UAS transgenes, these tester flies may have lower expression of caspase-LOV and display milder degeneration. There was no noticeable cell death 72 h after 10 min of illumination. We quantified the dendrite structures of control or Wld^S^-expressing c4da neurons at 24 or 72 h following 10 min of illumination or dark (Fig. 3 *A*–*D*). We found that 10 min of illumination resulted in shorter, fewer dendrites and a smaller percentage of territory covered in both control and Wld^S^-expressing neurons at 24 and 72 h afterward (Fig. 3 *A*–*D)*. Compared with control neurons, neurons with Wld^S^ expression exhibited increased dendrite length and tip numbers at 24 h after the illumination or maintenance in the dark and increased dendrite length at 72 h only after illumination (Fig. 3 *A*–*C*).

The dendrite dynamic analysis also revealed changes in dendrite length or tip numbers (Fig. 3 *E* and *F*); numbers of stable, eliminated, or added dendrite tips (Fig. 3 *G*–*I*); and the percentage of eliminated or added dendrites over a period of 48 h following 10-min caspase-LOV activation or maintenance in the dark (Fig. 3 *J* and *K*). For the changes over the 24- to 72-h period, 10 min of illumination reduced the increase in dendrite length, the dendrite tip number, and the number of stable or added dendrite tips in the control neurons kept in the dark (Fig. 3 *A*, *E*–*G*, and *I*). Neurons expressing Wld^S^ appeared to exhibit similar changes in dendrite length and tip numbers over the 24- to 72-h period when kept in the dark or with 10 min of illumination (Fig. 3 *A*, *E*, *F*, and *I*) and a decrease in the percentage of added dendrites when neurons were kept in the dark (Fig. 3 *A* and *K*). Interestingly, we found that compared with control, neurons expressing Wld^S^ retained significantly longer dendrites at 72 h (Fig. 3 *A* and *C*) and a lower percentage of eliminated dendrites (Fig. 3 *A* and *J*) upon 10 min of illumination. These protections provided by expression of Wld^S^ only occurred following 10 min of illumination but not when neurons were kept in the dark (Fig. 3 *A*, *C*, and *J*). These findings suggest that following caspase-LOV activation, Wld^S^ protected the dendrite structures by reducing dendrite degeneration, and this protection was dependent on the transient caspase-LOV activation.

### Caspase-LOV Activation of Different Durations in Adult Sensory Neurons Initiates Graded Dendrite Degeneration Followed by Repair or Cell Death.

Having found that dendrites of developing larval c4da neurons were capable of regrowth after transient caspase-LOV activation, we went on to test how mature c4da neurons would react to caspase-LOV–induced neurodegeneration. Unlike larval c4da neurons that continue to grow and form new dendritic branches during development, adult c4da neurons reach maturity around 3 d after eclosion and have stabilized dendrite structure throughout the rest of adulthood (8, 40, 41). Therefore, we decided to induce caspase-LOV activation in the mature adult sensory neurons at 7 d after eclosion and monitored the degeneration and regeneration thereafter to investigate how mature neurons respond to injury. From the results shown in Fig. 1, we learned that the chronic low-level caspase-LOV activity in the dark caused mild but significant dendrite degeneration during early larval development. To examine whether adult c4da neurons with low-level caspase activity in the dark can survive with regrowth of dendrites after metamorphosis, we imaged adult v’ada c4da neurons labeled with ppk-tdTOM and expressing caspase-LOV driven by ppk-GAL4 at 1 and 7 d after eclosion. We found that the mild degeneration of neurons expressing caspase-LOV without light exposure continued toward the adult stage (*SI Appendix*, Fig. S7). There were significant reductions in the survival rates of adult c4da neurons with low-level caspase activity in the dark at 1 and 7 d after eclosion (*SI Appendix*, Fig. 7*B*). The surviving neurons had impaired dendrite structures with significantly reduced dendrite length and dendrite tip number as compared with control c4da neurons without caspase-LOV at 1 and 7 d after eclosion (*SI Appendix*, Fig. S7 *A*, *C*, and *D*). We did not find any surviving neurons expressing caspase-LOV at 14 d after eclosion after surveying a total of 10 animals.

To reduce the early degeneration induced by the low-level caspase-LOV activity, we used a drug-inducible GeneSwitch ppk-GAL4 (ppk-GS) to drive the expression of the UAS–caspase-LOV, which would only be activated in the presence of the progesterone derivative RU486 (mifepristone) (42–46). Without drug induction, c4da neurons harboring ppk-tdTOM, UAS–caspase-LOV, and ppk-GS in wandering larvae had similar dendrite length and a slightly higher percentage of territory covered compared with control neurons labeled by ppk-tdTOM (*SI Appendix*, Fig. S8 *A*, *B*, and *D*). There was a significant but mild reduction in dendrite tip numbers (*SI Appendix*, Fig. S8 *A* and *C*). All adult c4da neurons harboring UAS–caspase-LOV and ppk-GS survived and regrew their dendrites after metamorphosis, with dendrite length and tip numbers comparable with those of control neurons at 1 d after eclosion without RU486 (*SI Appendix*, Fig. S8 *E*–*G*). At 7 d after eclosion, dendrites of both control neurons and neurons with UAS–caspase-LOV and ppk-GS continued to extend in length (*SI Appendix*, Fig. S8 *E* and *F*). Control neurons also increased their dendrite tip numbers (*SI Appendix*, Fig. S8 *E* and *G*). Neurons with UAS–caspase-LOV and ppk-GS exhibited small reductions in both dendrite length and tip numbers compared with control neurons at 7 d after eclosion (*SI Appendix*, Fig. S8 *E*–*G*). The degeneration that occurred without drug induction was likely due to the leaky expression previously reported for the GeneSwitch system (42, 47). For quantification of adult c4da neurons, we again built a deep learning model using a collection of adult c4da neurons generated in house to automatically segment the dendrite structure. Like our prediction model for larval c4da neurons, adult dendrite structures predicted by the adult model also had high Dice coefficients when compared with manual reconstruction (*SI Appendix*, Fig. S9 *A* and *B*). The nonoverlapping dendrites were mostly faint processes (*SI Appendix*, Fig. S9*B*). When we compared dendrite structure parameters derived from model-predicted segmentation after postprocessing with true answers from the manual reconstruction of images not included in the training dataset, we observed high correlations for both tip numbers (*R*^2^ = 0.99) and total dendrite length (*R*^2^ = 0.94) (*SI Appendix*, Fig. S9 *C* and *D*).

**Fig. 4.**
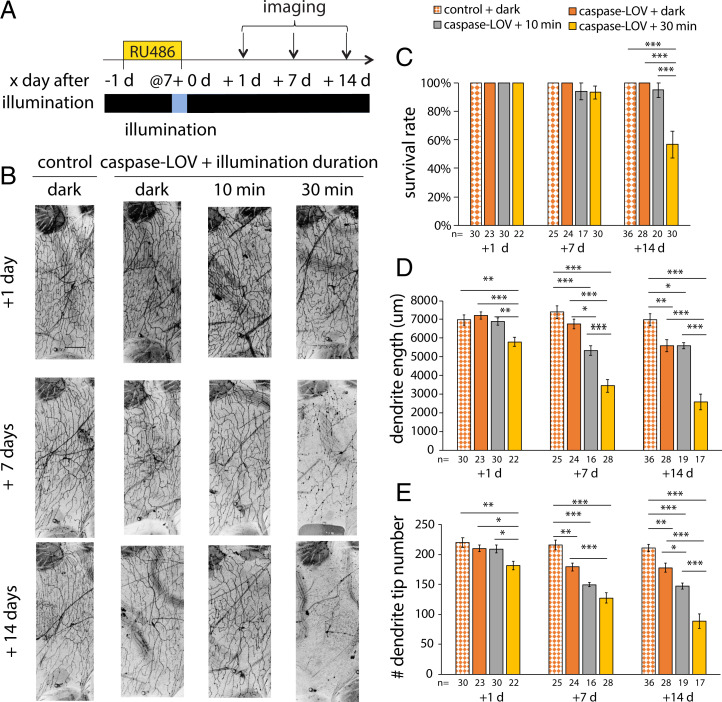
Illuminations induced graded dendrite degeneration in adult c4da neurons. (*A*) Protocol to feed RU486, illuminate, and image adult flies. Animals are raised in a normal light–dark cycle until moved to a food vial containing RU486 when flies are 6 d old. After a day with drugs, flies were illuminated for 10 or 30 min before being transferred back to normal food vials and kept in the dark. c4da neurons were imaged 1, 7, or 14 d after illumination. (*B*) Representative images of c4da neurons from adults harboring ppk-tdTOM, ppk-GS, and UAS–caspase-LOV and fed with EtOH (control) or RU486 (caspase-LOV) for a day before illumination. (Scale bar: 100 μm.) (*C*) Survival rates of c4da neurons at 1, 7, and 14 d after illumination. Significantly more neurons expressing caspase-LOV were found dead at 14 d after 30 min of illumination. (*D* and *E*) Quantifications of dendrite structures of survived c4da neurons, including total dendrite length (*D*) and total dendrite tip numbers (*E*). One-way ANOVA with Tukey’s post hoc test for multiple comparisons was used in *C*–*E*. Error bars represent ± SEM. *n* = 16 to 36 neurons for each experimental condition and time point as noted. *P < 0.05; **P < 0.01; ***P < 0.001.

To induce the expression of caspase-LOV, we transferred 6-d-old adult flies from normal food to food with 10 mM RU486 for a day before 10 or 30 min of illumination when the flies were 7 d old (Fig. 4*A*). Flies were returned to normal food and kept in the dark after the illumination. We imaged c4da neurons at 1, 7, and 14 d afterward to capture the early and late stages of caspase-3–induced dendrite degeneration and subsequent repair (Fig. 4*A*). Ten or thirty minutes of illumination by itself without caspase-LOV expression did not affect dendrite length and tip numbers of adult neurons at 1, 7, or 14 d after illumination (*SI Appendix*, Fig. S10).

Without illumination, adult c4da neurons expressing caspase-LOV induced by 1-d exposure of 10 mM RU486 remained alive for at least 14 d (Fig. 4 *B* and *C*). Neurons from flies treated with drugs and kept in the dark exhibited reduced dendrite length and tip number starting at 7 and 14 d, respectively, compared with neurons from control flies treated with ethanol (EtOH) and kept in the dark (Fig. 4 *B*–*D*). Only about 6 and 5% of neurons in flies treated with the drug were found dead at 7 and 14 d after 10 min of illumination, respectively (Fig. 4 *B* and *C*). When we illuminated drug-treated flies for 30 min, there were significantly fewer surviving neurons (57%) 14 d after illumination compared with neurons from flies illuminated for 10 min (95%) or kept in the dark (100%) (Fig. 4 *B* and *C*). Ten minutes of illumination resulted in significant decreases in the dendrite length and tip numbers starting at 7 d after illumination (Fig. 4 *B*, *D*, and *E*). For the neurons that survived 30 min of illumination, we observed significant reductions in dendrite length and tip numbers earlier, at 1 d after illumination, and the degeneration that continued over the following 2 wk was worse than that in control flies and flies expressing caspase-LOV without light exposure or exposure to light for 10 min (Fig. 4 *B*, *D*, and *E*). Taken together, our data suggest that mature neurons can also survive transient caspase-LOV activation and exhibited graded dendrite degeneration. The degree of degeneration varies with the duration of caspase-LOV activation controlled by illumination.

### Wld^S^ Can Reduce Caspase-LOV–Induced Dendrite Degeneration in Mature c4da Neurons.

**Fig. 5.**
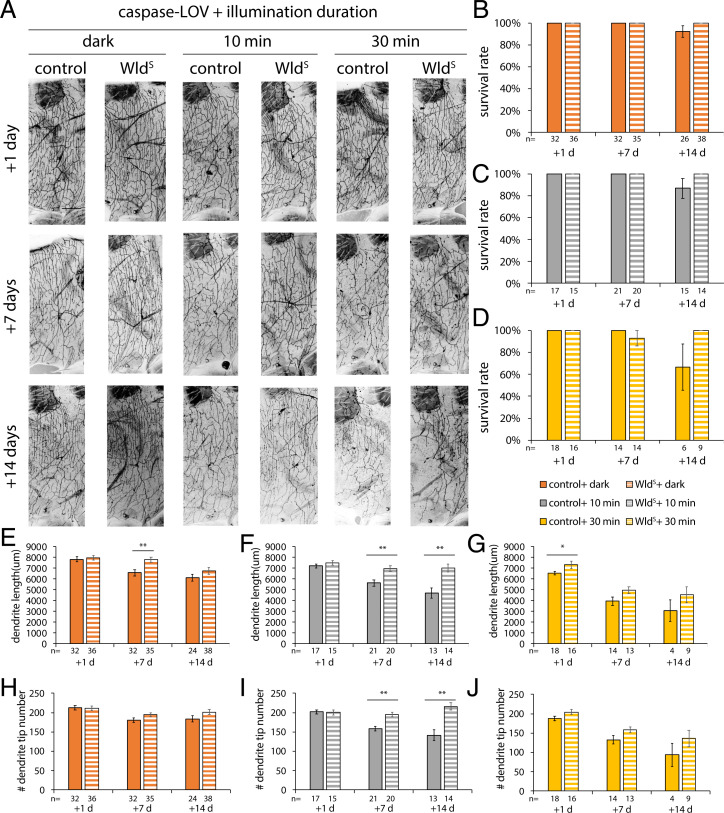
Expression of Wld^S^ can protect adult c4da neurons from mild dendrite degeneration. (*A*) Representative images of c4da neurons from adults harboring ppk-tdTOM, ppk-GS, UAS–caspase-LOV, and UAS-mIFP-2A-HO1 (control) or UAS-Wld^S^ (Wld^S^). Adult flies were fed with RU486 for a day before illumination at 6 d after eclosion. Animals were illuminated for 10 or 30 min and imaged following the protocol in Fig. 4A. (Scale bar: 100 μm.) (*B*–*D*) Survival rates of c4da neurons from animals kept in the dark (*B*), illuminated for 10 min (*C*), or illuminated for 30 min (*D*) were measured at 1, 7, and 14 d after illumination. (*E*–*J*) Quantifications of dendrite structures of survived c4da neurons from animals kept in the dark (*E* and *H*), illuminated for 10 min (*F* and *I*), or illuminated for 30 min (*G* and *J*). The measurements include total dendrite length (*E*–*G*) and total dendrite tip numbers (*H*–*J*). One-way ANOVA with Tukey’s post hoc test for multiple comparisons was used in *C*–*E*. Error bars represent ± SEM. *n* = 6 to 38 neurons for each experimental condition and time point as noted. *P < 0.05; **P < 0.01.

To test whether Wld^S^ expression can also reduce dendrite degeneration in adult c4da neurons as in the case of larval c4da neurons, we examined the dendrite structure of c4da neurons expressing ppk-tdTOM with caspase-LOV and Wld^S^ or mIFP-2A-HO1 (control) driven by ppk-GS from flies with a 1-d treatment of 10 mM RU486. Following the protocol described in Fig. 4*A*, we found that all neurons with Wld^S^ expression survived 10 or 30 min of illumination at 14 d after illumination, while 87 or 67% of control neurons were found dead after illumination for 10 or 30 min, respectively (Fig. 5 *A*–*D*). Without illumination, neurons with the expression of Wld^S^ retained longer dendrites 7 d after treating these flies with 10 mM RU486 (Fig. 5 *A* and *E*), while they showed no significant alteration in the dendrite structure at 1 and 14 d after treatment and without illumination (Fig. 5 *A*, *E*, and *H*). Upon 10 min of illumination, neurons expressing Wld^S^ displayed attenuated dendrite degeneration and retained longer dendrites and more dendrite tips at 7 and 14 d after illumination (Fig. 5 *A*, *F*, and *I*). Upon 30 min of illumination, Wld^S^ afforded protection in dendrite length at 1 d after illumination but not 7 or 14 d after illumination (Fig. 5 *A*, *G*, and *J*). Our data suggest that transient expression of Wld^S^ induced by 1 d of treatment of 10 mM RU486 can reduce dendrite degeneration following 10 min of illumination in mature c4da neurons for up to 2 wk. As for stronger caspase-LOV activation induced by 30 min of illumination, the protection by Wld^S^ expression was only observed at 1 d after illumination.

### Dendrite Regeneration following Caspase-LOV Activation in Adult c4da Neurons.

**Fig. 6.**
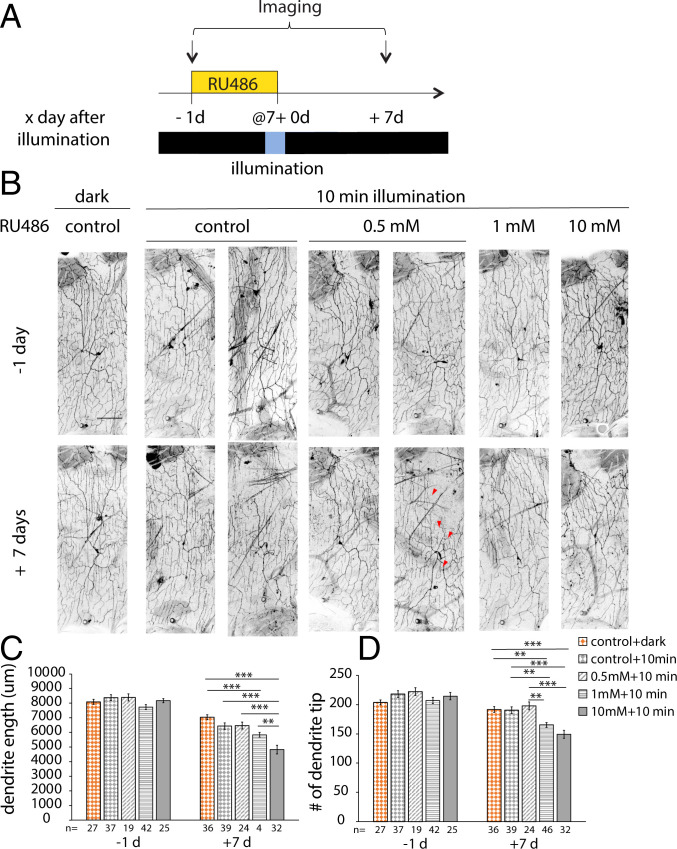
A low level of drug induction induced mild dendrite degeneration in the mature neurons. (*A*) Protocol to feed RU486, illuminate, and image adult flies. After a day with varying concentrations of drugs or EtOH (control), flies were kept in the dark or illuminated for 10 min before being transferred back to normal food vials and kept in the dark. c4da neurons were imaged 1 d before and 7 d after illumination. (*B*) Representative images of c4da neurons from adults harboring ppk-tdTOM, ppk-GS, and UAS–caspase-LOV and fed with EtOH (control) or RU486 (caspase-LOV) for a day before illumination. The red arrowheads indicate the regenerated dendrites in the c4da neurons 7 d after illumination. (Scale bar: 100 μm.) (*C* and *D*) Quantifications of dendrite structures of surviving c4da neurons, including total dendrite length (*C*) and total dendrite tip numbers (*D*). One-way ANOVA with Tukey’s post hoc test for multiple comparisons was used in *C* and *D*. Error bars represent ± SEM. *n* = 19 to 46 neurons for each experimental condition and time point as noted. **P < 0.01; ***P < 0.001.

Dendrite regeneration of adult c4da neurons following laser severing has been reported previously (8). However, it is unclear whether dendrites of mature neurons can regenerate following caspase-3–induced neurodegeneration. Following the experimental protocol in Fig. 4*A*, we observed little dendrite regeneration after 10 or 30 min of illumination on neurons in flies with a 1-d treatment of 10 mM RU486 (Figs. 4*B* and 5*A*). To test for the possibility that treatment with 10 mM RU486 might have resulted in inhibition of dendrite regeneration, we asked whether c4da neurons can regenerate dendrites in flies treated with 1 or 0.5 mM RU486. To monitor dynamic changes in dendrite degeneration and regeneration, we imaged the same neurons prior to the 1-d treatment with RU486 followed by illumination (−1 d) and 7 d after illumination (+7 d) (Fig. 6*A*). We found that 10 min of illumination on animals fed with 1 or 10 mM RU486 for a day induced mild but significant dendrite degeneration 7 d afterward as compared with control neurons without RU486 treatment (Fig. 6 *B*–*D*). The reductions in dendrite length induced by 10 mM RU486 treatment and 10 min of illumination were greater than the reductions induced by 1 mM RU486 treatment and 10 min of illumination (Fig. 6 *B*–*D*). Ten minutes of illumination on animals without drug induction or with 0.5 mM RU486 treatment for a day did not show statistical differences in dendrite length and tip numbers, even though adult c4da neurons did display local degeneration events over the course of 3 d following mild caspase-LOV activation (Fig. 6 *B*–*D* and *SI Appendix*, Fig. S11).

**Fig. 7.**
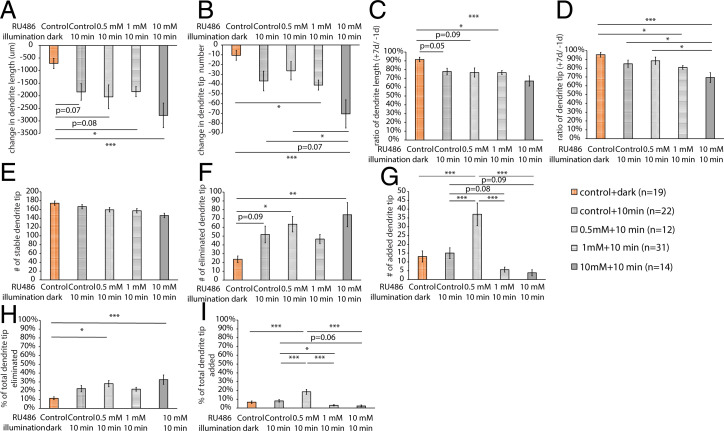
Adult c4da neurons can regenerate following the activation of lower expression of caspase-LOV. (*A* and *B*) Quantifications of changes in dendrite length (*A*) and dendrite tip numbers (*B*) of c4da neurons between 1 d before (−1 d) and 7 d after (+7 d) caspase-LOV activation. c4da neurons expressing caspase-LOV exhibited decreases in dendrite length and dendrite tip numbers with 10 min of illumination. (*C* and *D*) Quantifications of +7/−1 d ratios of dendrite length (*C*) and tip numbers (*D*) for the same neurons following 10 min of illumination. (*E*–*G*) Quantifications for the number of stable (*E*), eliminated (*F*), and added (*G*) dendrite tips over the −1- to +7-d period following caspase-LOV activation. (*H* and *I*) Quantifications for the percentage of eliminated (*H*) and added (*I*) dendrite tips over the −1- to +7-d period following caspase-LOV activation. One-way ANOVA with Tukey’s post hoc test for multiple comparisons was used in *A*–*I*. Error bars represent ± SEM. *n* = 12 to 31 neurons for each experimental condition and time point as noted. *P < 0.05; **P < 0.01; ***P < 0.001.

To quantify dendrite regeneration, we performed the dendrite dynamic analysis on neurons that were treated with EtOH (control) and kept in the dark and neurons that survived 10-min caspase-LOV activation and were treated with EtOH or 0.5, 1, or 10 mM RU486 over a period of 7 d following caspase-LOV activation (Fig. 7). The 10-min illumination on animals without drug induction had a trend in increasing the differences in dendrite length and the number of eliminated dendrites and showed a decrease in the +7/−1 d ratios of dendrite length (Fig. 7 *A*, *C*, and *F*). When the drug treatment was increased to 1 or 10 mM, we started to see significant increases in the changes in dendrite length and tip numbers and decreases in the +7/−1 d ratios of dendrite length and tip number following 10 min of illumination when compared with the control neurons kept in the dark (Fig. 7 *A*–*D*). We also found that 0.5 and 10 mM drug treatments were sufficient to induce significantly more dendrite elimination and a higher percentage of eliminated dendrite tips following 10 min of illumination (Fig. 7 *F* and *H*). The numbers of stable dendrites were similar between all conditions (Fig. 7*E*). Intriguingly, we observed a significant increase in the number and percentage of added dendrite tips in neurons treated with 0.5 mM RU486 following 10 min of illumination compared with all other groups of neurons (Fig. 7 *G* and *I*). This phenomenon of increasing the number and percentage of added dendrite tips is diminished when the drug treatment was increased from 0.5 to 1 mM (Fig. 7 *G* and *I*). Taken together, our data suggest that there was concurrent dendrite degeneration and regeneration following caspase-LOV activation in mature neurons over the 7 d after illumination, and the regenerative capacity of mature neurons diminished with increased caspase-LOV activity.

### Wld^S^ Does Not Improve Dendrite Regeneration following Caspase-LOV Activation in Adult c4da Neurons.

**Fig. 8.**
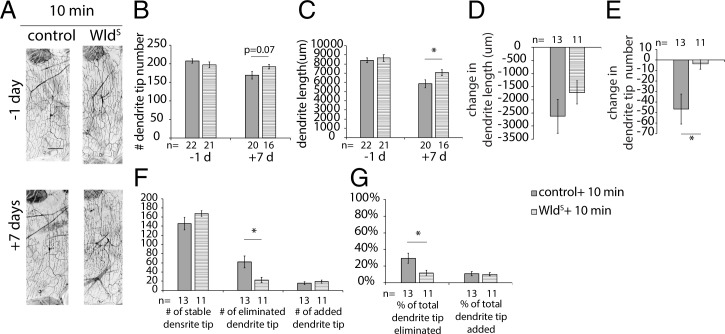
Expression of Wld^S^ decreased dendrite degeneration without changing dendrite regeneration. (*A*) Representative images of c4da neurons from adults harboring ppk-tdTOM, ppk-GS, UAS–caspase-LOV, and UAS-mIFP-2A-HO1 (control) or UAS-Wld^S^ (Wld^S^). Adult flies were fed with 1 mM RU486 for a day before illumination at 6 d after eclosion. Animals were illuminated for 10 min and imaged following the protocol Fig. 6A. (Scale bar: 100 μm.) (*B* and *C*) Quantifications of dendrite structures of c4da neurons, including total dendrite length (*B*) and total dendrite tip numbers (*C*). (*D* and *E*) Quantifications of changes in dendrite length (*D*) and dendrite tip numbers (*E*) of c4da neurons between 1 d before (−1 d) and 7 d after (+7 d) caspase-LOV activation. (*F*) Quantifications for the number of stable (*Left*), eliminated (*Center*), and added (*Right*) dendrite tips over the −1- to +7-d period following caspase-LOV activation. (*G*) Quantifications for the percentage of eliminated (*Left*) and added (*Right*) dendrite tips over the −1- to +7-d period following caspase-LOV activation. Student’s *t* test was used in *B*–*G*. Error bars represent ± SEM. *n* = 11 to 22 neurons for each experimental condition and time point as noted. *P < 0.05.

To test whether Wld^S^ expression can improve dendrite regeneration in adult c4da neurons, we examined the dendrite structure of neurons from flies expressing ppk-tdTOM with caspase-LOV and Wld^S^ or mIFP-2A-HO1 (control) driven by ppk-GS with a 1-d treatment of 1 mM RU486. Following the protocol described in Fig. 6*A*, we found that neurons with Wld^S^ expression retained significantly longer dendrites and exhibited a trend in increased dendrite tip numbers (*P* = 0.07) at 7 d after illumination (Fig. 8 *A*–*C*). Expression of Wld^S^ did not affect changes in dendrite length over 7 d following illumination (Fig. 8 *A* and *D*). We observed smaller reductions in dendrite tip numbers in neurons expressing Wld^S^ (Fig. 8 *A* and *E*). With the dendrite dynamics assay, we found that Wld^S^ expression significantly reduced the number of eliminated dendrite tips, while the numbers of stable and added dendrite tips were comparable with those of control neurons (Fig. 8 *A* and *F*). The percentage of the eliminated dendrite tips also decreased with Wld^S^ expression, whereas the percentage of the added dendrite tips remained unchanged (Fig. 8 *A* and *G*). These data suggest that Wld^S^ expression was sufficient to reduce dendrite degeneration in the mature c4da neurons, but it did not increase dendrite regeneration following caspase-LOV–induced dendrite degeneration.

## Discussion

In this study, we established an assay system for inducing neurodegeneration with the photo-switchable caspase-3, caspase-LOV, to elucidate the mechanisms underlying dendrite degeneration and repair in both developing larval and mature adult neurons. To characterize the caspase-3–induced neurodegeneration, we focused on the dendrite morphology for c4da neurons and observed graded dendrite degeneration in both larval and adult c4da neurons depending on the degree of caspase-LOV activation. We found that Wld^S^, a key molecule involved in the Wallerian axon degeneration, can reduce dendrite degeneration and cell death caused by caspase-LOV activation. Our data suggest that the protection afforded by Wld^S^ is through reducing the percentage of dendrite elimination but not through increasing dendrite regrowth or dendrite regeneration. Our assay with adjustable caspase-LOV activation provides a useful platform to search for genes involved in neurodegeneration and repair so as to shed light on strategies to prevent neurodegeneration, diagnose neurodegeneration early, and develop drugs promoting neural recovery from injury and diseases.

### Advantages of the Assay System for Caspase-3–Induced Neurodegeneration.

The range of dendrite degeneration and repair resulting from varying degrees of caspase-LOV activation demonstrates the versatility of the photo-switchable caspase-3 system to induce degeneration in *Drosophila* da neurons with multiple dendrites. This assay system has several strengths. First, photo-switchable caspase-3 granted us control over the activity of caspase-3. In this study, we demonstrated that we could control the degree of degeneration by adjusting the duration of illumination. Because of the photo-switchable feature of caspase-LOV, we can control the onset of degeneration by adjusting the start time of illumination. We can also pharmacologically control the expression level of caspase-LOV with the GeneSwitch drug-inducible system employed in our adult assay. These temporal controls over the extent of caspase-LOV expression and activation provide the potential to examine the alteration of neuronal responses with aging for comparison with previous findings with the laser-ablation model or glaucoma (8, 31). Second, in contrast to laser severing of dendrites, the photo-switchable caspase-3 allows for infliction of neuronal injury in a way that is considerably less labor intensive. It is thus amenable to screens of genetic manipulations or pharmacological drug libraries to dissect the underlying cellular and molecular mechanisms. Third, the caspase-LOV assay system is likely to provide physiologically relevant insights given that caspase-3 plays a role in the developmental pruning of axon and dendrites, injury-induced neurodegeneration, and neurodegenerative diseases (15–21). Our data show that activation of caspase-LOV can induce mild degeneration in both the primary and tertiary branches. This pattern of dendrite degeneration has been described as the dendrite pathology in some of the injury and disease models (1–5). This is distinct from the laser ablation with the severing of primary branches. Therefore, our assay system can complement the existing injury or neurodegeneration models and provide an additional platform to study how to repair dendrites following neurodegeneration.

### Mechanistic Studies Enabled by the Caspase-LOV System.

In this study, the dendrite elimination observed in the c4da neurons following caspase-LOV activation is likely a mix of dendrite retraction and degeneration. This kind of complex neurite elimination process without cell death has been observed during normal development, as in the case of degeneration of larval neuronal dendrites followed by pruning before the elaboration of dendrites in pupal stages as well as in response to injury or diseases in the adult (34, 38, 48). For c4da neurons, local dendrite degeneration includes a series of dendrite destruction programs, such as severing of proximal dendrites, thinning and blebbing of disrupted branches, dendrite branch fragmentation, and removal of the dendritic debris (19, 32, 34, 38, 39, 49). It will be interesting to use the recently developed time-lapse imaging protocol to follow the progress of degeneration and repair in the larval neuron to further characterize the dendrite elimination events following the caspase-LOV activation (39).

Following caspase-LOV activation, c4da neurons underwent graded dendrite degeneration, protection, repair, or apoptosis for both developing and mature neurons. In this paper, we refer to dendrite degeneration, protection, and repair as operational terms. We define protection as reducing a spectrum of deficiencies induced by caspase-LOV activation, which includes cell death, reduced dendrite structure, reduced dendrite addition, and increased dendrite elimination. Thus, the protection results in increased cell survival and/or retaining longer dendrites and more numerous dendrite tips. Repair is the process that happened following caspase-LOV activation in the surviving neurons with active growth, although we cannot exclude the possible involvement of changes in normal dendrite growth. Regeneration is defined as an increase in dendrite length or tip number in response to the caspase-LOV–induced degeneration excluding developmental dendrite growth. Adult neurons only have limited normal growth, so the newly added branches we identified following caspase-LOV–induced degeneration in the mature adult neurons mostly reflect regeneration in response to the degeneration (8, 40, 41). Because dendrite addition in larvae could be a mix of regeneration and changes in normal dendrite growth, we described regeneration only for the mature adult c4da neurons (7 d after eclosion). Future studies of the underlying cellular and molecular mechanisms may help to determine to what extent these events examined in our study are similar to conventional neurodegeneration, protection, and repair.

With parallel studies of developing and mature neurons, we can aim for a more comprehensive understanding of dendrite degeneration and repair and can determine whether there are shared or unique gene functions important for dendrite degeneration and repair across different developmental stages. For example, the prorepair genes identified in the larval system could be improving developmental growth, injury-dependent regrowth, or both. The adult system, on the other hand, would help us to focus on injury-dependent regeneration and can serve as a platform to examine whether candidate genes identified in the larval neurons can affect the regenerative capacities of mature neurons. Using the caspase-LOV system, we can monitor the progression of dendrite degeneration by imaging neurons at multiple time points. In the larval neurons, we tracked the degeneration and repair and imaged neurons at 24 and 72 h after photo-activation of caspase-LOV. In the adult system, we could follow dendrite degeneration and repair for 7 to 14 d. It is possible to follow the neurons for a longer period if desired. Given that acute and continuing responses to the insults are both critical hallmarks for developing therapeutic strategies for neurodegeneration, future studies may examine the functions of candidate genes at various time points during the degeneration and repair processes.

### The Protection Afforded by Wld^S^ May Vary with the Degree of Neurodegeneration.

The Wallerian degeneration pathway is important for axon degeneration and has served as a prominent target for therapy (26, 27). In addition to validating the intriguing involvement of Wld^S^ in dendrite degeneration that has been previously reported (28–32), we found that expression of Wld^S^ can ameliorate varying degrees of dendrite degeneration in the developing and mature neurons, but the protection in the mature neurons experiencing strong caspase-LOV activation is only transient (Fig. 8 *F*, *G*, *I*, and *J*). This may explain why Wld^S^ fails to rescue the survival of flies with neuronal expression of caspase-LOV with days of continuous activation of the caspase-LOV (22), a much stronger perturbation compared with conditions used in this study.

Because Wld^S^ protection of dendrite degeneration may vary with the levels of caspase-LOV activation, we speculate that there are different sets of genes involved in these varieties of responses upon caspase-LOV–induced neurodegeneration. For instance, there could be genes involved in the repair of mild but not severe degeneration or genes that play roles in neuronal survival but not in dendrite degeneration specifically. Candidate genes can also exert opposite functions during the degeneration and repair processes. Studies have started to show that some candidate genes, including nicotinamide mononucleotide adenylyltransferase and Dual leucine zipper kinase, that were found to protect neurons from degeneration and cell death may prohibit the capacity to regenerate (50, 51). Notably, *Wld^S^* mice also exhibit poor axon regeneration with delayed axon degeneration (52).

### Cell Type–Specific Cellular Responses upon Caspase-3–Induced Dendrite Degeneration.

There are four different classes of da neurons for sensory transduction. Extensive studies in da neurons have revealed cell type–specific dendrite morphology, gene expressions, dendrite remodeling, and injury responses (6, 7, 12, 14, 40). In the current study, we examined how c4da neurons react to caspase-LOV activation because they have the most complex dendrites among the da neurons (7). It would be of interest to survey whether there is any cell type–specific mechanism for caspase-3–induced dendrite degeneration in individual types of da neurons. In conjunction with the genetic tools available, we could induce degeneration in specific cell types. By elucidating the limitations and potentials of different classes of da neurons in response to neurodegeneration, we could assess the cell type–specific programs that can be transferred to other cell types to maximize their ability to withstand the caspase-LOV–induced neurodegeneration and improve their capacity to repair in each class of da neurons. Even though we focus on cell-autonomous factors in this study, we recognize there are likely noncell-autonomous contributions from epidermal cells and glial cells (8, 12, 53–55). These noncell-autonomous contributions could be addressed by incorporating another regulation over gene expression in other cell types while expressing caspase-LOV in the c4da neurons.

### Possible Improvements of the Caspase-LOV Assay System.

The assay system we introduced in this study has expanded our understanding of dendrite degeneration and repair. There are several potential avenues for improving this assay system, such as the inclusion of independent control over the expression of candidate genes apart from the caspase-LOV.

For both adult and larval systems, expressions of the caspase-LOV and transgenes are controlled together by ppk-GAL4 or ppk-GS. To determine the time of action of candidate genes, future improvements for better temporal control of the overexpression of transgenes could make use of other inducible gene expression systems, such as the LexA/LexAop and the Q system, to test out the ideal innervation time to induce the expression of transgenes (56, 57). Developing parallel systems to control the expression of candidate genes is also important to investigate adult regeneration. Given that c4da neurons can only regenerate following minimum expression of caspase-LOV, using the same control over candidate genes may yield only subtle effects from the candidate genes.

Previous work has reported that illumination can significantly increase the activity of caspase-LOV compared with their activity assayed in the dark (22). Notably, the activity of caspase-LOV in the dark can be significantly reduced by the C450A mutation of caspase-LOV that renders caspase-LOV in the inactive conformation (22). These data suggest that caspase-LOV kept in the dark exhibits some basal and sustained caspase activity at a lower level than the transient boost of caspase-LOV activity upon illumination. From our data, we found that c4da neurons can withstand the activity of basal caspase-LOV in the dark through larval development and were able to make it into adulthood before starting to degenerate early in adulthood. In this study, we focused on how neurons react to the neurodegeneration and repair induced by transient mild caspase-LOV activation. In future work, it will be interesting to look at how the larval neurons managed to withstand the low but constant basal caspase activity and how larval and adult neurons respond differently to the constant low basal caspase activity vs. the transient high caspase activity.

Since the low-level caspase-LOV activity in the dark can induce mild degeneration in the background with illumination, it will be desirable to find a variant of caspase-LOV with reduced leakage in the dark perhaps by protein engineering. It will also be interesting to adopt other photo-switchable handles to control the activity of the caspase-3 (58, 59). For example, it is possible to substitute the LOV domain with the Dronpa domain, which can be turned on with 405 Ultraviolet (UV) light and turned off by 488-nm blue light (60, 61). Another layer of control to switch off the caspase-3 through light may assure the termination of the caspase-3 activity.

## Materials and Methods

### Fly Stocks.

Animals were reared at 25 °C unless otherwise indicated. *SI Appendix* has details of the fly stocks used in this study.

### Adult GeneSwitch Drug-Inducible Caspase-LOV Expression.

To activate GeneSwitch-mediated expression of caspase-LOV, adult flies were housed in food vials with 50 μL of 0.5, 1, or 10 mM RU486 Mifepristone (Sigma; M8046) dissolved in EtOH or just EtOH as controls. RU486 was added to the surface of food and air dried at room temperature for 1 d before use. After a day in the vial with the drug, animals were moved back to normal yeasted food vials for the rest of the experiment.

### Illumination Protocol.

To activate the photo-switchable caspase-3, freely moving larvae were picked at 48 h after egg laying (AEL) and transferred to the transparent and yeasted agar plates for different durations of blue light emitting diode (LED) illumination. Larvae were moved back to the yeasted grape plate and kept in the dark at 22 °C afterward. We lowered raising temperature to 22 °C to delay development and increase the temporal resolution of the repair process following caspase-3 activation. To illuminate adult c4da neurons, we mount the flies in a custom-built chamber with water to keep them moist and leave the flies in the chamber under the blue LED light. *SI Appendix* has details of the illumination setups.

### In Vivo Time-Lapse Imaging.

To image larval and adult c4da neurons, we follow the protocol described before, which allows us to follow changes in individually identified neurons in vivo at multiple time points (8, 14). *SI Appendix* has details.

### Segmentation and Quantification of Dendrite Structure.

We utilized two methods to segment the dendrite structures of the da neurons for morphological quantification. For larval c4da neurons, in Fig. 1, we reconstructed individual neurons using Vaa3D-Neuron 2.0: 3D neuron paint and tracing function in Vaa3D (https://alleninstitute.org/what-we-do/brain-science/research/products-tools/vaa3d/) with manual correction and validation of the tracing (62). For the rest of the larval and adult c4da neurons in this study, we established a U-Net–based deep learning model for automatic dendrite structure segmentation, which produces segmentation maps with pixel intensity representing the probability of dendrite structure. With the manually constructed dendrite structure or skeletal images predicted by the deep learning models, we can obtain the total dendrite length, dendrite tip numbers, percentage of territory covered, and dendrite complexities assessed with Sholl analysis. *SI Appendix* has details.

### Software.

The code used for deep learning–based automatic dendrite structure prediction is written in Python/TensorFlow. The software package, training, and example testing images are available in the GitHub repository (https://github.com/chienhsiang/dendrite_U-Net). *SI Appendix* has details.

### Statistical Tests.

All data are presented as mean ± SEM based on at least three independent experiments. Data are considered significantly different when *P* values are less than 0.05. We did statistical analysis between all groups. When two groups were not connected by a line, that means there were no differences between them. Statistics analysis was performed and prepared using JASP (version 0.14) or in the R environment. All samples were prepared and analyzed in parallel. *SI Appendix* has details.

## Supplementary Material

Supplementary File

## Data Availability

The software package, training, and example testing images are available in the GitHub repository (https://github.com/chienhsiang/dendrite_U-Net) (63). All other data are included in the article and/or *SI Appendix*.
