# Supplementary material for: A photo-switchable assay system for dendrite degeneration and repair in Drosophila melanogaster
Source: Proc Natl Acad Sci U S A. 2022 Aug 15;119(34):e2204577119. doi: 10.1073/pnas.2204577119 (PMC9407391; doi:10.1073/pnas.2204577119)
Supplement: Supplementary File [file pnas.2204577119.sapp.pdf]

PNAS

Supplementary Information for

A photo-switchable assay system for dendrite degeneration and repair in *Drosophila melanogaster*

Han-Hsuan Liu[abc], Chien-Hsiang Hsu[d], Lily, Y. Jan[abc] and Yuh-Nung Jan[abc*]

[a] *Department of Physiology, University of California, San Francisco, CA 94143;*
[b] *Department of Biochemistry and Biophysics, University of California, San Francisco, CA 94143;*
[c] *Howard Hughes Medical Institute, University of California, San Francisco, San Francisco, CA 94143;*
[d] *Department of Pharmaceutical Chemistry, University of California, San Francisco, San Francisco, CA 94143;*
* Yuh-Nung Jan
Email: yuhnung.jan@ucsf.edu

This PDF file includes:

SI Materials and Methods
Figures S1 to S11
SI References

Supplementary Information Text

SI Materials and Methods

Fly stocks

The fly strains used in this study were as follows: UAS-Wld^S (a generous gift from Ashley Smart at UCSF (1)), GAL4$^{19-12}$ (2), GAL4$^{2-21}$ (3), ppk-GAL4 (4), ppk-CD4-tdGFP (5), ppk-tdTOM (BL35844) UAS-caspase-LOV (BL76355, a generous gift from Ashley Smart at UCSF), UAS-tdTomato (5), UAS-mIFP-T2A-HO1 (insertion site is attp40 on 2nd chromosome with wild-type, w^{1118} background. a generous gift from Xiaokun Shu, UCSF), ppk-GS (a generous gift from Rebecca Yang at Duke University Medical Center, NC, USA and Jay Parrish at University of Washington, WA, USA (6)). The tester line for larval experiments was ppk-gal4, ppk-CD4-tdGFP; UAS-caspase-LOV. The tester line for adult experiments was ppk-CD4-tdTOM; ppk-GS, UAS-caspase-LOV.

Illumination setups

For illuminating larvae, we used a homemade 40 cm x 10 cm x 15 cm carbon box to house three 30 cm long and 8mm wide Blue 3528 LED strips, (single row of 60 LEDs per meter, Environmental Lights) stick on the ceiling of the box in parallel and connected by wires. The power of the light 15 cm away from the LED strips, where larvae were kept, is 0.91 mW/cm^2.

For illuminating adult flies, three 7.5 cm long and 10 mm wide Blue 3528 LED strips, (single row of 240 LEDs per meter, Environmental Lights) were attached to a piece of glass in parallel and connected by wires. This piece of glass with LED strips was kept 2 cm away from the flies during illumination. To maintain the temperature, we install aluminum heat sinks with a 120x120x38mm 24V DC industrial cooling case fan (Wathai) turned on throughout the illumination period. The power of the light 2 cm away from the LED strips, where adult flies were kept, is 29.5 mW/cm^2.

In vivo time-lapse imaging

Live imaging of larvae was performed as described before (7). Larvae were anesthetized with diethyl ether for 5-8 minutes (Acros Organics) before being mounted in glycerol on top of a thin patch of agarose. For adult flies, we use the carbon dioxide source as an anesthetic throughout the imaging sessions. After images were acquired using a Leica SP5 microscope with a 20X oil objective (NA 0.75), larvae or flies were returned to yeasted grape juice agar plates or yeasted food vials. Sum slices for Z-projection were generated using ImageJ software and used for dendrite structure prediction as described later.

Deep learning based-automatic dendrite structure prediction

For the deep learning based-model for automatic dendrite structure segmentation, we followed the U-Net architecture specified in the original study (8) by modifying the channel number of the final segmentation map from 2 to 1 since we only predicted dendrite structure versus background. Each training data consisted of a maximum intensity Z-projection image of one neuron manually cropped by drawing an ROI, paired with the manually segmented dendrite structure (mask) generated using the plugin, “simple neurite tracer”, in ImageJ. For the larval model, we generated 29 sets of image-mask pairs for training and 8 sets for validation. For the adult model, we generated 65 sets of image-mask pairs for training and 17 sets for validation. These datasets were all generated in-house.

We trained our model on a Quadro P5000 Graphics processing unit (GPU) with 16 GB random-access memory (RAM) in a Dell Precision 7920 Tower with Dual Intel Xeon Gold 6136 central processing units (CPUs) (3.0/3.7 GHz), having 12 cores and 128 GB RAM. The operating system was Windows 10. We have tested our system on Mac and Windows operating systems. Two data augmentation strategies were used to increase the robustness of our model. First, an area of 512x512 pixels was randomly cropped from each input 1024x1024 training image and the associated mask. Then the cropped image and mask were randomly flipped horizontally and vertically with a probability of 0.5. We used the sum of binary cross-entropy and Dice loss (defined as 1 – Dice coefficient) as the loss function and trained the model with Adam optimizer at learning rate 1e-4 for 500 epochs. The best model evaluated by Dice loss using the validation dataset was chosen for the downstream analysis. Our larval model achieved the Dice loss at 0.13 and 0.16 for

training and validation datasets, respectively and our adult model achieved the Dice loss at 0.12 and 0.17 for training and validation datasets, respectively.

A threshold of 0.5 was used to binarize segmentation maps generated by the model. We found a high correlation (R^2 = 0.98) in the total dendrite length of larval neurons between model-predicted segmentation and manual reconstruction, while tip numbers only showed a moderate correlation (R^2 = 0.45). This was because tip number was more sensitive to discontinuity and small fragments occasionally found in model-predicted segmentation masks. Therefore, we included a 3-step post-processing procedure to exclude small fragments and reduce the discontinuity in the segmented dendrite structure. First, small objects with areas less than 10 pixels (7.75 μm) were discarded. Second, dilation with a cross-shaped structuring element (connectivity=1) was used to fill in the gaps. Finally, skeletonization using the *skeletonize* function from Python scikit-image package was applied to obtain the final segmentation for the downstream morphology quantification. With post-processing to fill in gaps and remove small fragments, we observed a dramatic increase in the correlation of tip numbers (R^2 = 0.97) and a slight increase in total dendrite length (R^2 = 0.99). The post-processing procedure also greatly improve the correlation of tip numbers (R^2 = 0.78 to R^2 = 0.99) and total dendrite length (R^2 = 0.98 to R^2 = 0.94) for the predictions made with adult models.

This deep learning based-automatic dendrite structure prediction system can be applied to predict the structures of other types of neurons using the exiting models or applied to create models with a new set of training datasets as long as one can separate the individual neurons at the manual ROI selection step.

Quantification of dendrite structure

Using the skeletal images, we performed Sholl analysis of dendrite branches to determine the complexity of the dendrite structure. The crossing continuous circles were separated by 0.76μm on either manually traced or predicted dendrite arbors. To determine the percentage of territory covered, we measured the territory covered by the dendrite arbor of the neuron of interest using ROI selection tools in ImageJ and divided it into the total area of the hemisegment of the body wall. We defined a cell as "survived" if the neuron has more than 2 tips (at least more than one neurite) and if the average dendrite length (total dendrite length/total tip numbers) is over 10 pixels (7.75 μm) to filter out small fragments that mostly contributed by the remain axons or debris. To reduce the batch-by-batch variations, we normalized the quantifications to the controls for each batch before combining all data. For comparison between different conditions, the number was normalized to the averaged number in dark (control). The results are normalized to the controls for each set of experiments before combining.

Statistical Tests

The Student's *t* test was used to compare between two groups. One-way ANOVA with Tukey's post hoc test was used for comparisons of multiple groups. The Kruskal–Wallis rank sum test with Dunn's post hoc test further adjusted by the Benjamini–Hochberg False Discovery Rate (FDR) method was used for multiple comparisons of nonparametric samples. Two-way ANOVA with Tukey's post hoc test was used for comparisons of interaction between two factors.

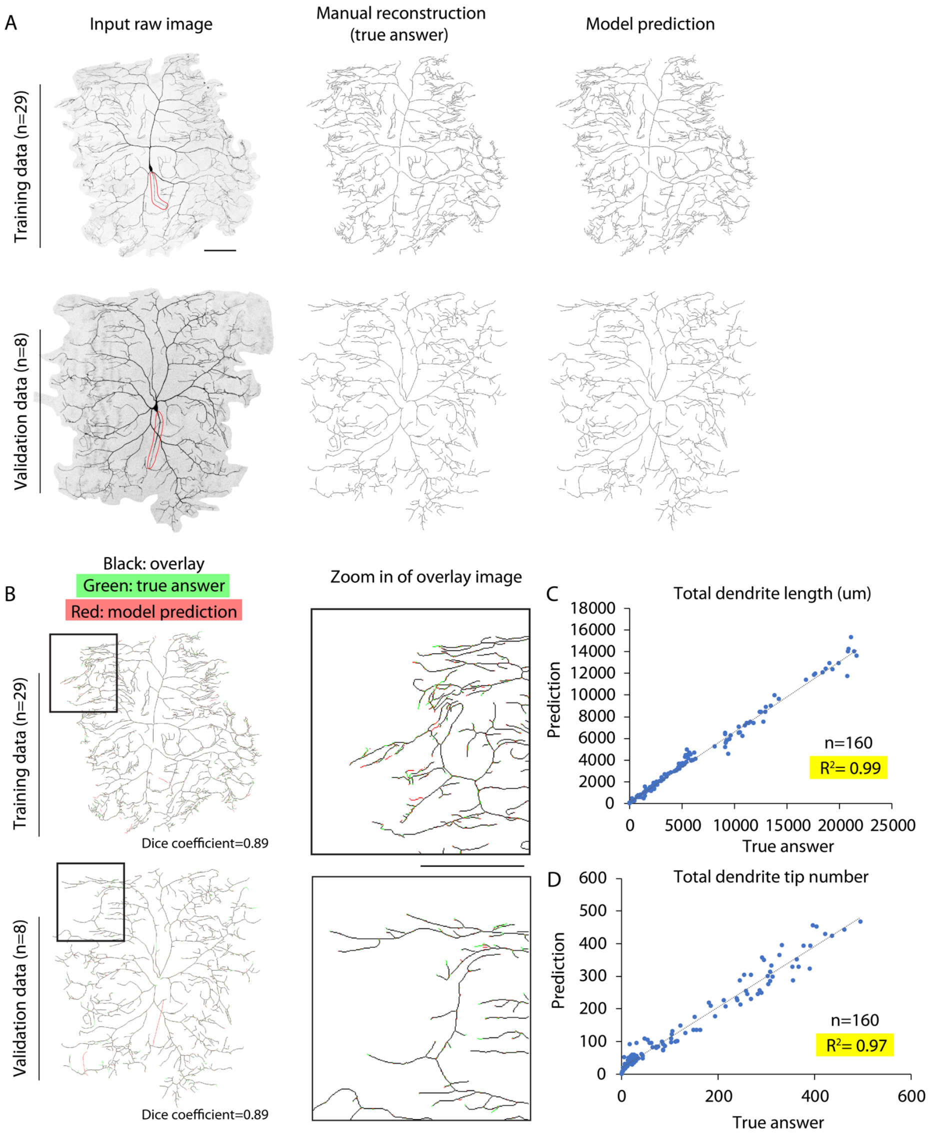

Fig. S1. Deep learning-based automatic dendrite structure prediction for larval c4da neurons. (A) Our in-house trained deep learning-based model performed well in dendrite segmentation. In the top row are images of a representative neuron from the training dataset and the bottom row is a neuron from the validation dataset (novel neurons for the model). The first column contains the input Z-projection image of neurons manually cropped by drawing an ROI. Images in the second column are manually segmented dendrite structure (true answer) from the ImageJ plugin, "simple neurite tracer". Our model predictions are in the third column. (C) The overlay and zoom-in images from true answer and model prediction are shown in (A). The model reliably recognized most of the arbors as human as most of the dendrites are matched (marked in black) with few distal dim dendrites omitted by the model and only shown in the true answer (green) or only recognized by the model (red). Our model did not differentiate between axons and dendrites and sometimes counts the axon (circled with red dash line in the first column) as one of the dendrites (7 out of 37 neurons in the training and validation dataset). (C-D) Relationships between manual reconstruction (true answer) and the deep learning model (prediction) for total dendrite length and total dendrite tip number of 160 neurons derived from manual reconstruction acquired in Fig. 1, which were not included in the training dataset. After post-processing, our prediction model achieved 0.99 for R^2 of total dendrite length (C) and 0.97 for R^2 of tip numbers (D). Scale bars =100 μm.

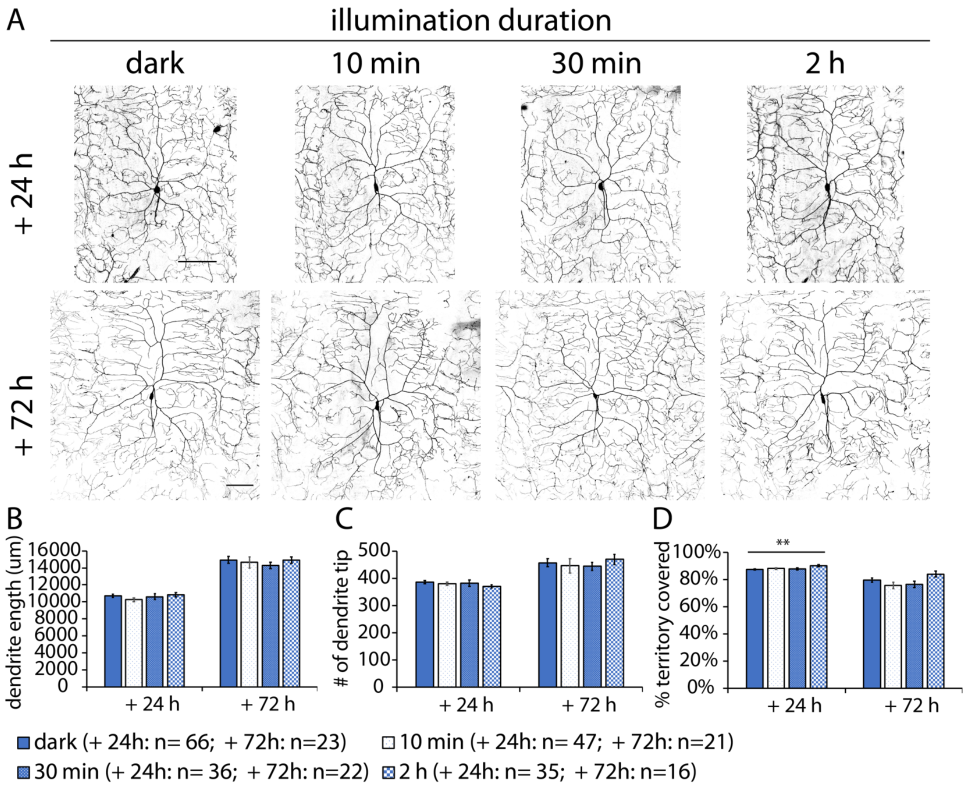

Fig. S2. Blue light illumination does not affect dendrite structures of larval c4da neurons. (A) Representative images of c4da neurons from larva without caspase-LOV and illuminated kept in the dark (control), with caspase-LOV and kept in the dark (dark), or with caspase-LOV and illuminated for 10 min, 30 min, and 2 h. Neurons were imaged at 24 h (+ 24 h, top row) and 72 h (+ 72 h, bottom row) after illumination started according to the protocol in Fig. 1A. (B-D) Quantifications of dendrite structures of survived c4da neurons following caspase-LOV activation, including total dendrite length (B), total dendrite tip numbers (C), and percentage of territory covered (D). Scale bars =100 μm. one-way ANOVA with Tukey's post hoc test for multiple comparisons in (B-D). Error bars represent ± SEM. n = 16-23 neurons for each experimental condition and timepoint as noted in the figure.

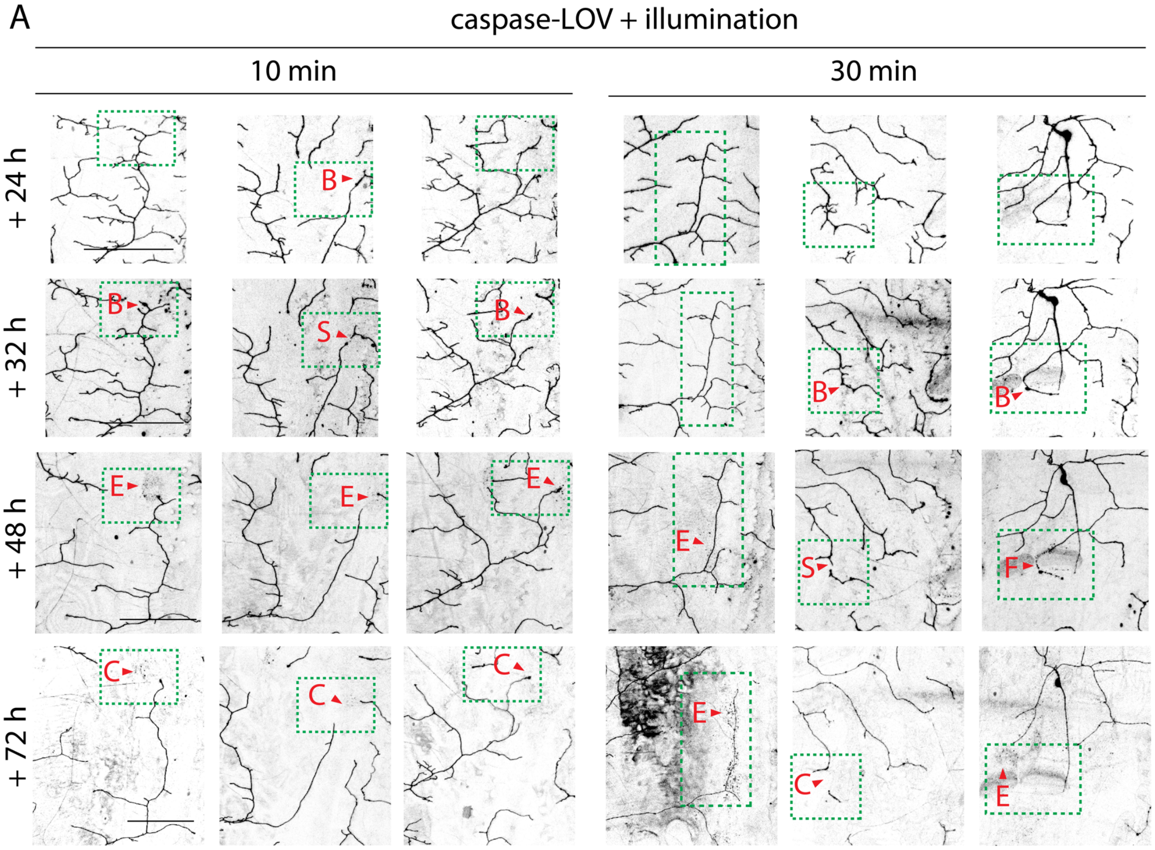

Fig. S3. Dendrite degeneration in larval c4da neurons following mild caspase-LOV activation. (A) Time course of dendrite degeneration of c4da neurons with caspase-LOV and labeled by CD4-tdTom following 10 min (3 columns on the left) or 30 min (3 columns on the right) illumination. The same neuron was imaged at 24 h, 32 h, 48 h, and 72 h after illumination started (from top to the bottom row). We observed local degeneration events following 10 min or 30 min illumination. Local degeneration included dendrite branch severing (S), dendrite branch fragmentation (F), dendrite blebbing (B), dendritic debris clearing (C), and engulfment of dendritic debris after breakdown (E) as noted next to the arrowheads. The sites of local degeneration were indicated by red arrowheads. The same region of dendrite arbors compared over time was outlined with a dashed green line. At least one of the local degeneration events was observed in 36 out of 38 neurons following 10 min illumination or 24 out of 24 neurons following 30 min illumination. Scale bars =100 μm.

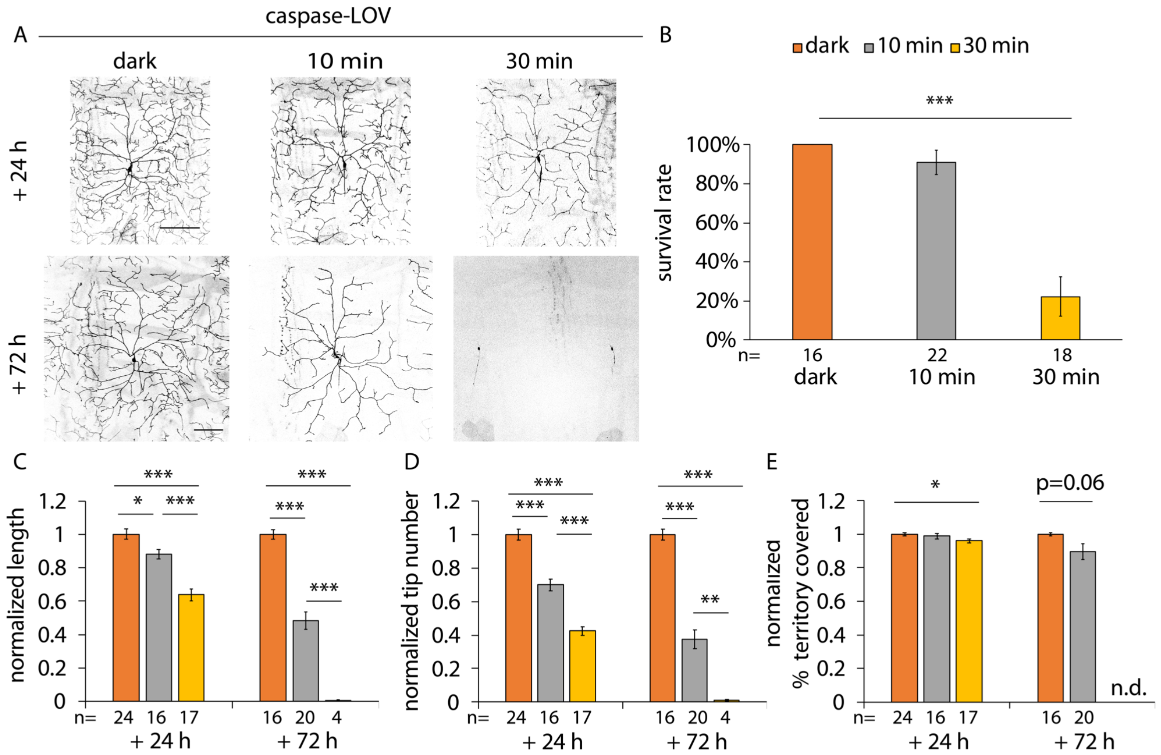

Fig. S4. Degeneration and repair in the c4da neurons of tester animals. (A) Representative images of c4da neurons expressing UAS-luciferase and UAS-caspase-LOV driven by ppk-gal4 and labeled with ppk-tdGFP. Larvae were illuminated for 10 min or 30 min and imaged following the protocol in Fig. 1A. (B) Survival rates of c4da neurons decreased significantly upon 30 min illumination. (C-E) Quantifications of dendrite structures, including normalized length (C), normalized tip numbers (D), and normalized percentage of territory covered (E) of c4da neurons kept in the dark, illuminated for 10 min or illuminated for 30 min. The dendrite degeneration in the surviving c4da neurons is worse when illumination is extended. Scale bars =100 μm. * $p<0.05$, ** $p<0.01$, *** $p<0.001$, Kruskal-Wallis rank-sum test with Dunn's post hoc test further adjusted by the Benjamini-Hochberg FDR method for multiple independent samples (B); one-way ANOVA with Tukey's post hoc test for multiple comparisons in (C-E). Error bars represent ± SEM. n = 16-24 neurons for each experimental condition and timepoint as noted in the figure.

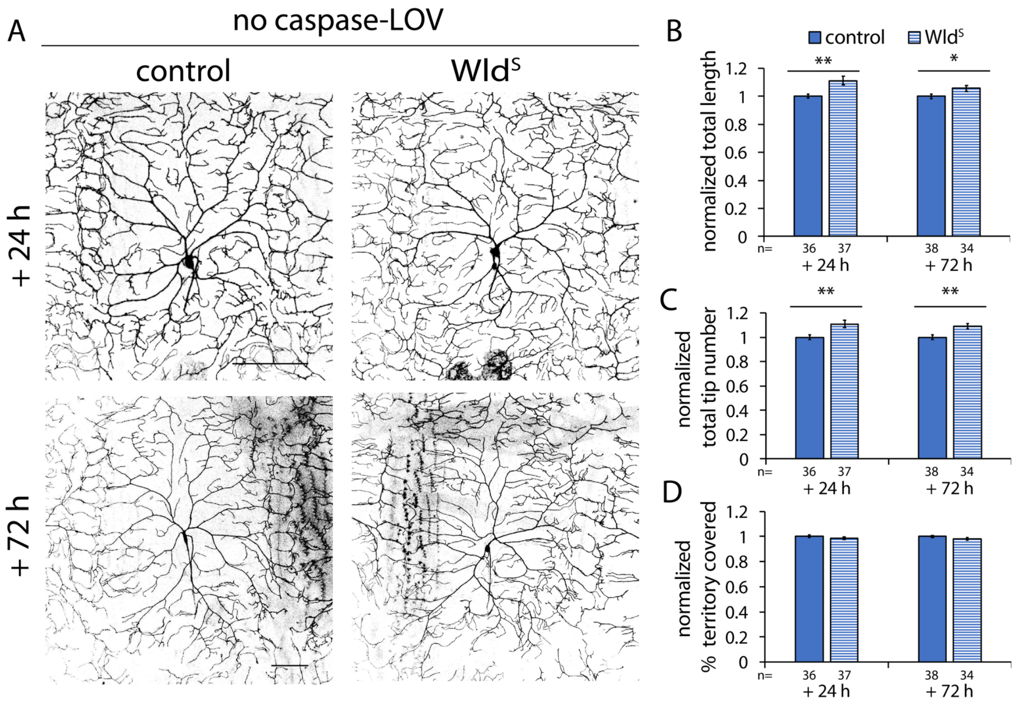

Fig. S5. Wld^S expressing neurons had longer and more dendrites during development. (A) Representative images of c4da neurons labeled by ppk-tdGFP with a ppk-Gal4 driving expression of UAS-mIFP-2A-HO1 (control) or UAS-Wld^S (Wld^S). Larvae were imaged following the protocol in Fig. 1A. (B-D) Quantifications of dendrite structures, including normalized length (B), normalized tip numbers (C), and normalized percentage of territory covered (D) of c4da neurons. Scale bars =100 μm. * $p<0.05$, ** $p<0.01$, *** $p<0.001$, Student's t-test in (B-D); Error bars represent ± SEM. n ≥ 34 neurons for each experimental condition and timepoint as noted in the figure.

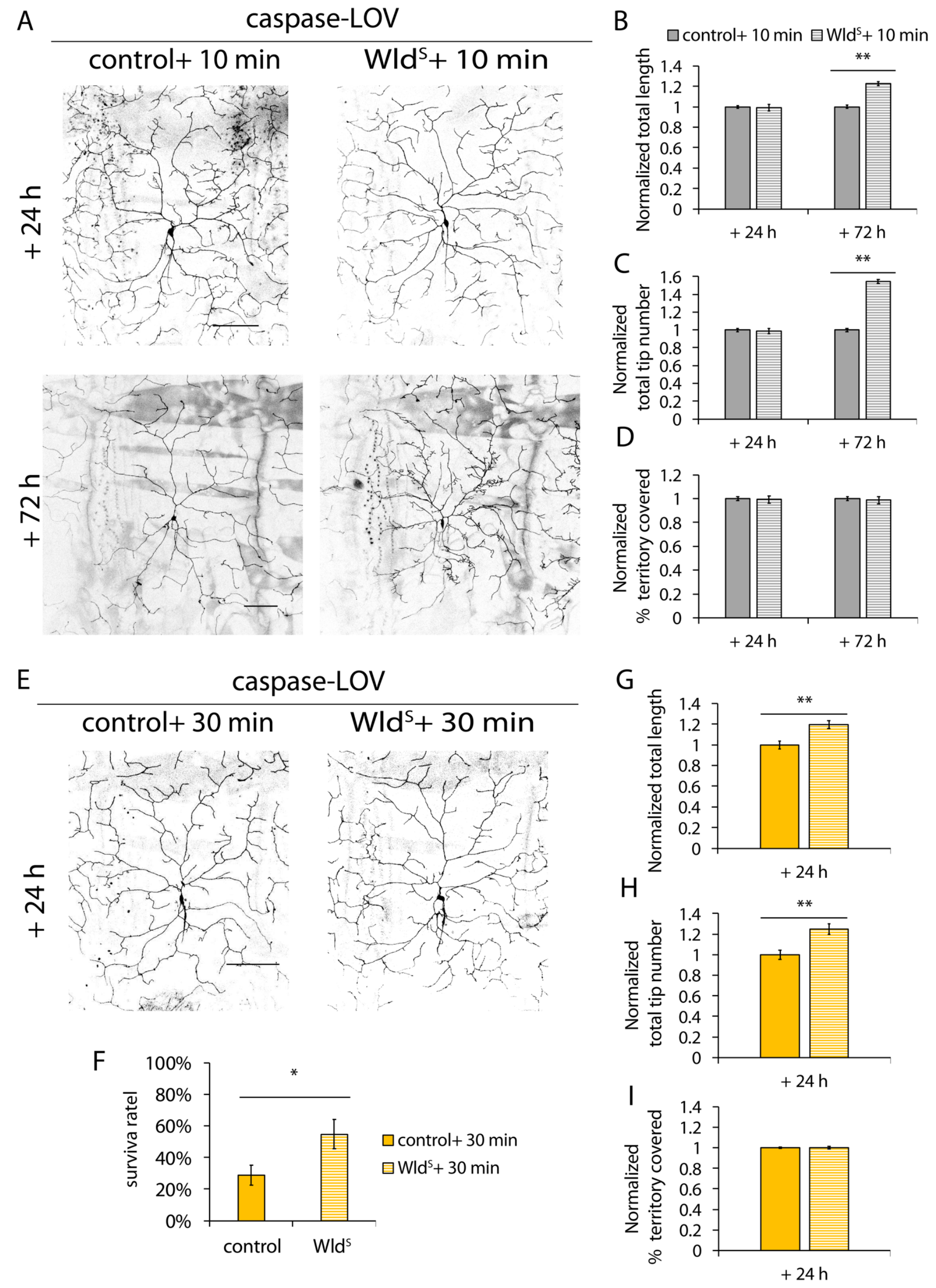

Fig. S6. Wld^S expressing neurons retain longer and more dendrites upon caspase-3 induced neurodegeneration. (A, E) Representative images of c4da neurons labeled with ppk-tdGFP and expressing caspase-LOV, and mIFP-2A-HO1 (control) or UAS-Wld^S (Wld^S) are driven by ppk-Gal4. Larvae were kept in the dark and illuminated for 10 min (A) or 30 min (E) at 48 h AEL and imaged 24 h or 72 h afterward. (B-D) Quantifications of dendrite structures, including normalized length (B), normalized tip numbers (C), and normalized percentage of territory covered (D) of c4da neurons illuminated for 10 min. (F-I) Quantifications of survival rate (F) and dendrite structures, including normalized length (G), normalized tip numbers (H), and normalized percentage of territory covered (I) of c4da neurons illuminated for 30 min. Scale bars =100 μm. * $p<0.05$, ** $p<0.01$, *** $p<0.001$, Student's t-test in (B-D, G-I), Kruskal-Wallis rank-sum test with Dunn's post hoc test further adjusted by the Benjamini-Hochberg FDR method for multiple independent samples (F); Error bars represent ± SEM. n ≥ 29 neurons for each experimental condition and timepoint as noted in the figure.

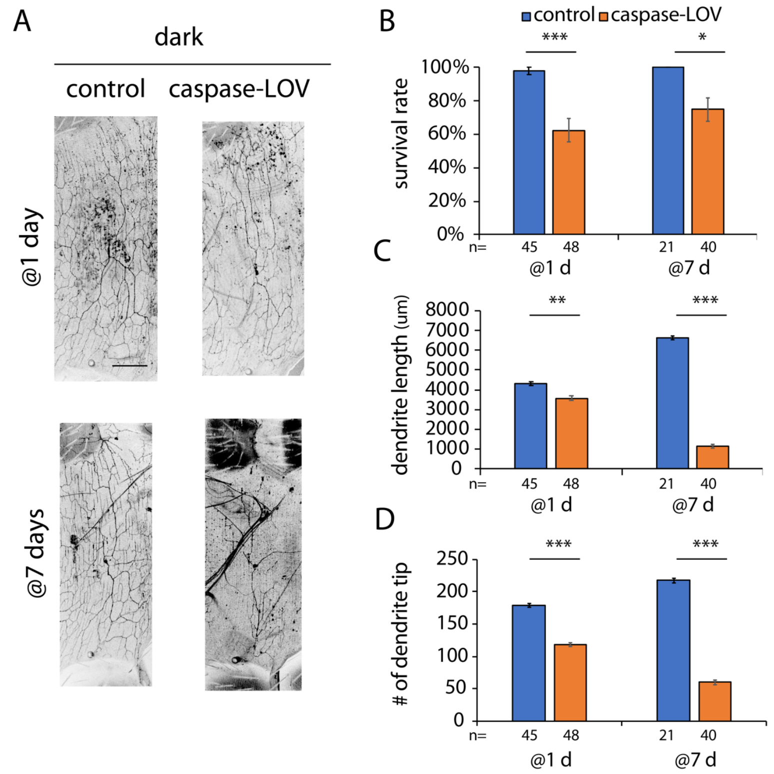

Fig. S7. The chronic low-level caspase-LOV activity resulted in early-onset neurodegeneration in adult c4da neurons. (A) Representative images of c4da neurons from adult flies harboring ppk-tdTOM and ppk-GAL4 (control), or adult flies harboring ppk-tdTOM, ppk-GAL4, and UAS-caspase-LOV. Animals were kept in the dark throughout and imaged at 1 day or 7 days after eclosion (@1 and @7). (B) Expression of caspase-LOV decreased survival rates of adult c4da neurons even when kept in the dark. (C-D) Quantifications of dendrite structures of @1 and @7 survived c4da neurons, including total dendrite length (C) and total dendrite tip numbers (D). Scale bars =100 μm. * $p<0.05$, ** $p<0.01$, *** $p<0.001$, Student's t-test in (B-D). Error bars represent ± SEM (C-F). n = 21-68 neurons for each experimental condition and timepoint as noted in the figure.

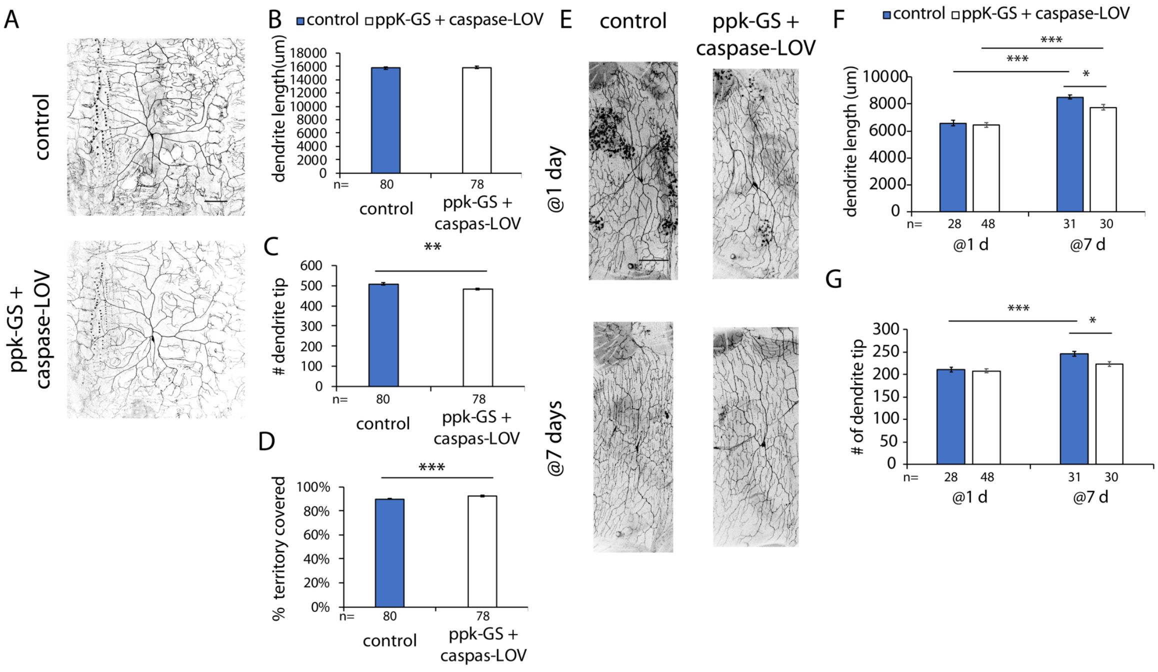

Fig. S8. Drug-inducible GAL4 gated the caspase-LOV expression and ameliorate the early onset neurodegeneration. (A) Representative images of c4da neurons from wandering larva harboring ppk-tdTOM (control), or ppk-tdTOM, ppk-GS, and UAS-caspase-LOV. Larvae were kept in a normal light-dark cycle without drug induction. (B-D) Quantifications of dendrite structures of survived c4da neurons following caspase-LOV activation, including total dendrite length (B), total dendrite tip numbers (C), and percentage of territory covered (D). Representative images of c4da neurons from adults harboring ppk-tdTOM (control), or ppk-tdTOM, ppk-GS, and UAS-caspase-LOV. Larvae were kept in a normal light-dark cycle without drug induction and imaged @1 or 7 days after eclosion. (F, G) Quantifications of dendrite structures of @1 and @7 survived c4da neurons, including total dendrite length (F) and total dendrite tip numbers (G). Scale bars =100 μm. * $p<0.05$, ** $p<0.01$, *** $p<0.001$, Student's t-test in (B-D); one-way ANOVA with Tukey's post hoc test for multiple comparisons in (F, G). Error bars represent ± SEm. n = 80 neurons for the control group and 78 neurons for the ppk-GS+ UAS-caspase-LOV group in (A-D) or 28-48 neurons in (E-G) for each experimental condition and timepoint as noted in the figure.

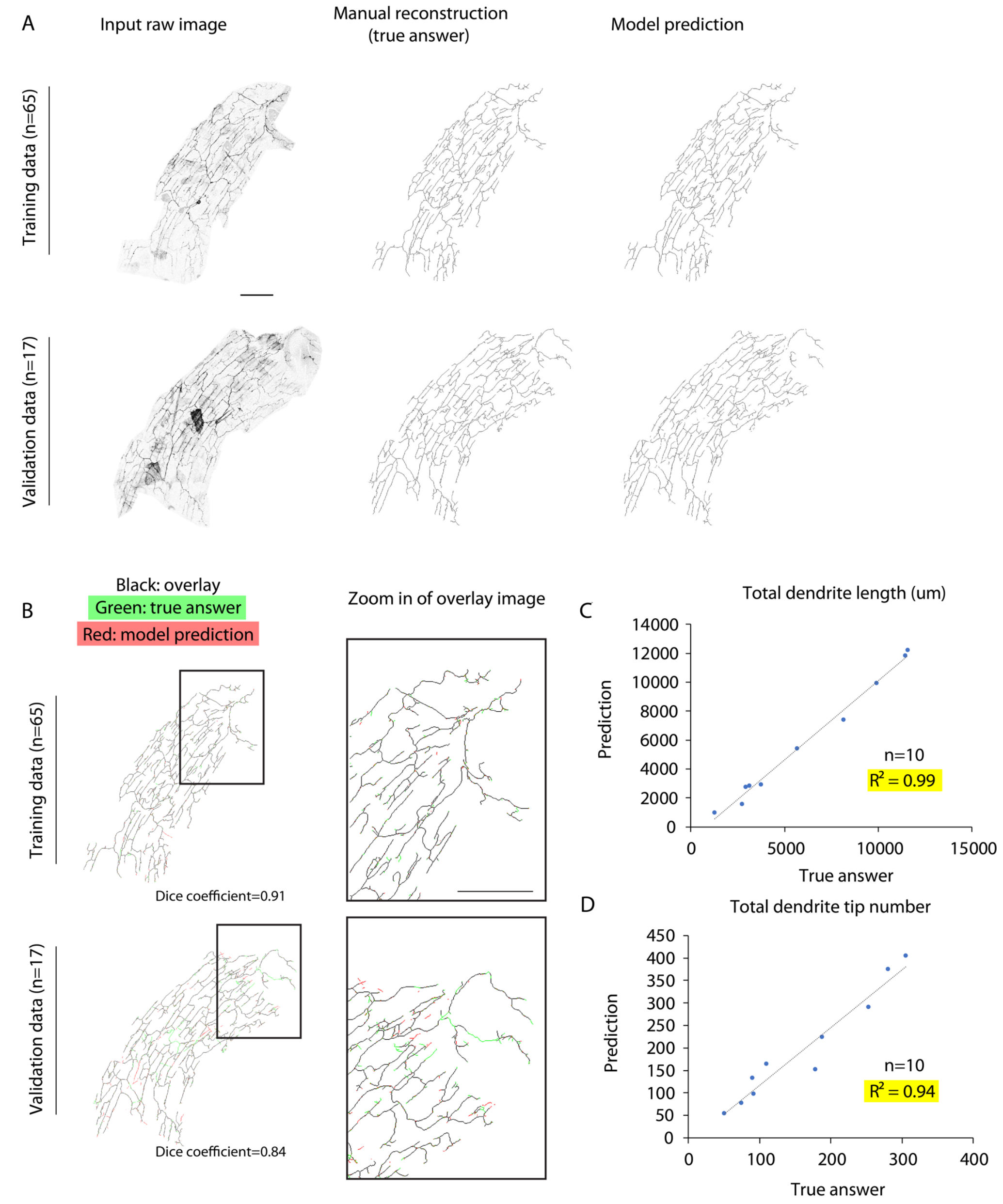

Fig. S9. Deep learning-based automatic dendrite structure prediction for adult c4da neurons. (A) Representative images of input images, manual reconstructions, and model predictions. In the top row are images of a representative neuron from the training dataset and the bottom row is a neuron from the validation dataset (novel neurons for the model). The first column contains the input Z-projection image of neurons manually cropped by drawing an ROI. Images in the second column are manually segmented dendrite structure (true answer) from the ImageJ plugin, “simple neurite tracer”. Our model predictions are in the third column. (C) The overlay and zoom-in images from true answer and model prediction are shown in (A). The model reliably recognized most of the arbors as human as most of the dendrites are matched (marked in black) with few distal dim dendrites omitted by the model and only shown in the true answer (green) or only recognized by the model (red). (C-D) Relationships between manual reconstruction (true answer) and the deep learning model (prediction) for total dendrite length and total dendrite tip number. After post-processing, our prediction model achieved 0.99 for R^2 of total dendrite length (C) and 0.94 for R^2 of tip numbers (D). Scale bars =100 μm. n = 10 neurons.

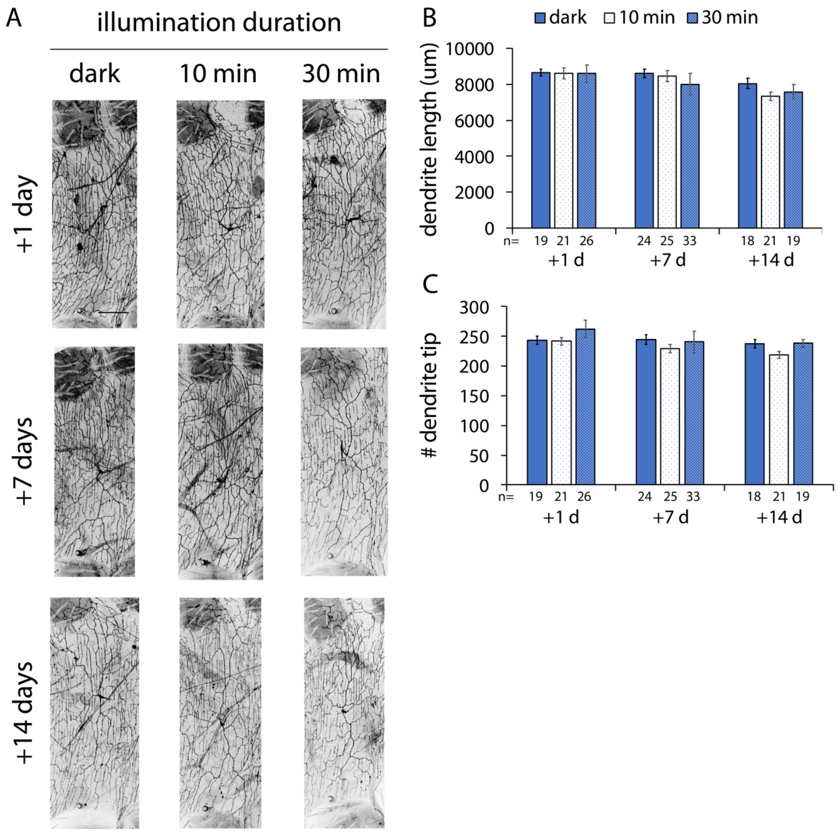

Fig. S10. Blue light illumination does not affect the dendrite structures of adult c4da neurons. (A) Representative images of c4da neurons from adults harboring ppk-tdTOM. Animals were illuminated for 10 min or 30 min and imaged following the protocol in Fig. 7A. (B, C) Quantifications of dendrite structures of c4da neurons, including total dendrite length (B) and total dendrite tip numbers (C). Scale bars =100 μm. * $p<0.05$, ** $p<0.01$, *** $p<0.001$, one-way ANOVA with Tukey's post hoc test for multiple comparisons in (B, C). Error bars represent ± SEM. n = 18-33 neurons for each experimental condition and timepoint as noted in the figure.

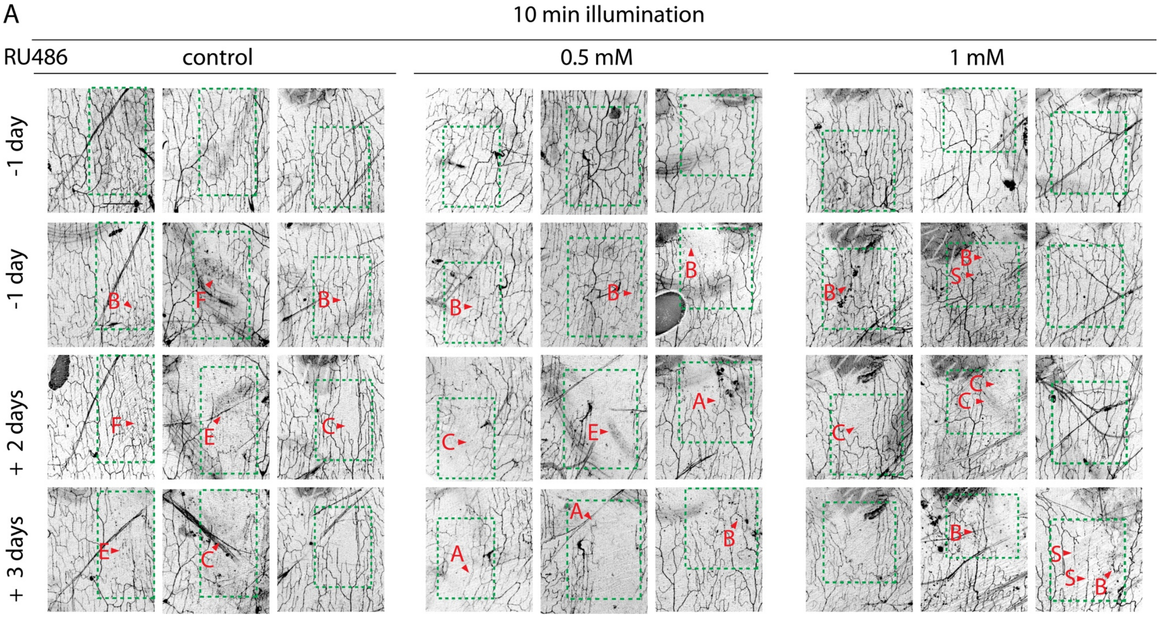

Fig. S11. Dendrite degeneration in adult c4da neurons following mild caspase-LOV activation. (A) Time course of dendrite degeneration of c4da neurons with ppk-GS and UAS-caspase-LOV and labeled by ppk-CD4-tdTom following 10 min illumination with EtOH (control, 3 columns on the left), 0.5 mM RU486 (3 columns in the middle), or 1 mM RU486 (3 columns on the right). The same neuron was imaged before drug treatment (-1 d) and at 1-3 d after illumination started (from top to the bottom row). We observed different local degeneration events following 10 min illumination in the control group and the group with mild drug treatment. We also observed dendrite regeneration in neurons treated with 0.5 mM RU486. The sites of local degeneration were indicated by red arrowheads and the dendrite addition (A) was marked by red solid arrowheads. Local degeneration included dendrite branch severing (S), dendrite branch fragmentation (F), dendrite blebbing (B), dendritic debris clearing (C), and engulfment of dendritic debris after breakdown (E) as noted next to the red arrowheads. The same region of dendrite arbors compared over time was outlined with a dashed green line. Scale bars =100 μm.

SI References

1. E. D. Hoopfer, et al., Wlds protection distinguishes axon degeneration following injury from naturally occurring developmental pruning. Neuron 50, 883–895 (2006).
2. Y. Xiang, et al., Light-avoidance-mediating photoreceptors tile the Drosophila larval body wall. Nature 468, 921–6 (2010).
3. W. B. Grueber, L. Y. Jan, Y. N. Jan, Different levels of the homeodomain protein cut regulate distinct dendrite branching patterns of Drosophila multidendritic neurons. Cell 112, 805–18 (2003).
4. W. B. Grueber, B. Ye, A. W. Moore, L. Y. Jan, Y. N. Jan, Dendrites of distinct classes of Drosophila sensory neurons show different capacities for homotypic repulsion. Curr Biol 13, 618–26 (2003).
5. C. Han, L. Y. Jan, Y.-N. Jan, Enhancer-driven membrane markers for analysis of nonautonomous mechanisms reveal neuron-glia interactions in Drosophila. Proc. Natl. Acad. Sci. U. S. A. 108, 9673–9678 (2011).
6. S. Rumpf, S. B. Lee, L. Y. Jan, Y. N. Jan, Neuronal remodeling and apoptosis require VCP-dependent degradation of the apoptosis inhibitor DIAP1. Dev. Camb. Engl. 138, 1153–1160 (2011).
7. K. L. Thompson-Peer, L. DeVault, T. Li, L. Y. Jan, Y. N. Jan, In vivo dendrite regeneration after injury is different from dendrite development. Genes Dev 30, 1776–89 (2016).
8. O. Ronneberger, P. Fischer, T. Brox, U-Net: Convolutional Networks for Biomedical Image Segmentation. ArXiv150504597 Cs (2015).
